# Characterization of the Poplar Pan-Genome by Genome-Wide Identification of Structural Variation

**DOI:** 10.1093/molbev/msw161

**Published:** 2016-08-07

**Authors:** Sara Pinosio, Stefania Giacomello, Patricia Faivre-Rampant, Gail Taylor, Veronique Jorge, Marie Christine Le Paslier, Giusi Zaina, Catherine Bastien, Federica Cattonaro, Fabio Marroni, Michele Morgante

**Affiliations:** ^1^Istituto di Bioscienze e Biorisorse, Consiglio Nazionale delle Ricerche, Sesto Fiorentino, Firenze, Italy; ^2^Istituto di Genomica Applicata (IGA), Udine, Italy; ^3^Dipartimento di Scienze Agro-alimentari, Università di Udine, Ambientali e Animali (DI4A), Udine, Italy; ^4^INRA US 1279 EPGV/CEA/CNG, Evry, France; ^5^School of Biological Sciences, University of Southampton, Southampton, United Kingdom; ^6^INRA, UR 0588 AGPF, Centre INRA Val de Loire, Orléans, France; ^7^IGA Technology Services (IGATS), Udine, Italy

**Keywords:** structural variation, pan-genome, transposable elements, poplar

## Abstract

Many recent studies have emphasized the important role of structural variation (SV) in determining human genetic and phenotypic variation. In plants, studies aimed at elucidating the extent of SV are still in their infancy. Evidence has indicated a high presence and an active role of SV in driving plant genome evolution in different plant species.

With the aim of characterizing the size and the composition of the poplar pan-genome, we performed a genome-wide analysis of structural variation in three intercrossable poplar species: *Populus nigra, Populus deltoides*, and *Populus trichocarpa*. We detected a total of 7,889 deletions and 10,586 insertions relative to the *P. trichocarpa* reference genome, covering respectively 33.2 Mb and 62.9 Mb of genomic sequence, and 3,230 genes affected by copy number variation (CNV). The majority of the detected variants are inter-specific in agreement with a recent origin following separation of species.

Insertions and deletions (INDELs) were preferentially located in low-gene density regions of the poplar genome and were, for the majority, associated with the activity of transposable elements. Genes affected by SV showed lower-than-average expression levels and higher levels of d*N*/d*S*, suggesting that they are subject to relaxed selective pressure or correspond to pseudogenes.

Functional annotation of genes affected by INDELs showed over-representation of categories associated with transposable elements activity, while genes affected by genic CNVs showed enrichment in categories related to resistance to stress and pathogens. This study provides a genome-wide catalogue of SV and the first insight on functional and structural properties of the poplar pan-genome.

## Introduction

Studies aimed at elucidating the genetic bases of complex phenotypes focused mostly on identifying genetic association between a given phenotype and single-nucleotide polymorphisms (SNPs). However, many studies have demonstrated that a substantial proportion of the heritability of complex traits cannot be explained by associations with SNPs (Vineis and Pearce 2010). Several researchers addressed the problem of “missing heritability” (Eichler et al. 2010) and suggested several causes, including rare variants (Manolio et al. 2009; Marroni et al. 2011) and structural variation (SV) (Eichler et al. 2010). It is now accepted that in addition to SNPs, other types of sequence variation play an important role in plant genome evolution (Bennetzen and Wang 2014; Marroni et al. 2014). Structural variation, such as insertions and deletions (INDELs) and copy number variation (CNV), has been shown to be frequent in plant species and to have an important effect on phenotypic diversity and genome evolution (Zmieńko et al. 2013; Marroni et al. 2014; Saxena et al. 2014).

Transposable element (TEs) activity is a major source of genome instability and structural variation. TE movement may directly mobilize gene sequences in the genome (Morgante et al. 2007) and may impact gene expression via the introduction or the modification of alternative regulatory elements, exons, and splice junctions (Ray and Batzer 2011) or via the modification of the local chromatin environment. A well-known example of the latter case has been reported in sweet orange, where the insertion of the *Rider* long terminal repeat (LTR) retrotransposon upstream of the Ruby gene resulted in its cold-dependent expression and in the distinctive red coloration of the variety Tarocco (Butelli et al. 2012). Moreover, TEs can mediate genome rearrangements through non-homologous recombination (Eichler and Sankoff 2003).

When CNVs change the number of copies of a given gene, they can alter their levels of expression. For example, gene amplification has been involved in stress resistance, resistance to herbicides, or tolerance to chemicals (Gaines et al. 2010; McHale et al. 2012; Maron et al. 2013).

The ubiquity of SV led some researchers to extend the pan-genome concept to plants (Morgante et al. 2007). According to this view, the pan-genome of a given species can be separated in a “core” genome, composed by sequences that are shared by all the species members and a “dispensable” genome, containing sequences that are present only in a subset of the individuals of that species. Sequences belonging to the dispensable genome, such as those involved in SV, may provide an important contribution to phenotypic diversity within the species (Marroni et al. 2014; Zhang et al. 2015).

In spite of its phenotypic relevance and its potential effects on genome evolution, SV has not been studied as carefully as other classes of sequence variants. The compact genome size (∼500 Mb) and the availability of the reference genome of *Populus trichocarpa* (Tuskan et al. 2006) make poplar a suitable model genus to study the prevalence of structural variation and its possible contribution to phenotypic variation. A better understanding of the population distribution of INDELs and CNVs might increase the ability to identify potentially interesting markers for poplar genetic improvement. Moreover, considering that different poplar species are crossable and progenies show hybrid vigor (Einspahr and Benson 1964; Li et al. 1993), a map of interspecific SV may be of help in elucidating the mechanisms at the basis of plant heterosis.

We set out to explore the extent of structural variation in the three poplar species *P. nigra*, *P. deltoides*, and *P. trichocarpa* at a genome-wide level by means of Illumina next-generation sequencing. The advent of NGS technologies has revolutionized the way of detecting SV and resequencing-based approaches have progressively replaced those based on microarrays (Alkan et al. 2011). The identification of SV using NGS data is mainly pursued by using two different approaches: (a) paired-end mapping (PEM), which identifies SV by using the discordance from the expected span size and/or orientation of mapped paired-end reads; and (b) depth of coverage (DOC), which detects SV by searching for a local increase or decrease in sequence depth (Alkan et al. 2011). We exploited the PEM signature to identify INDELs in the resequenced individuals with respect to the *P. trichocarpa* reference genome and the DOC signature to detect genic CNVs between all the resequenced samples. We studied how SV is distributed along the genome, its relationship with the activity of transposable elements and its possible impact on gene expression. Finally, we combined the information on all the detected variants to get a first estimate of the size and the composition of the poplar pan-genome.

## Results and Discussion

Studies on SV in plant species could have tremendous utility in identifying genomic regions associated with complex traits, domestication, and adaptation. To date, studies on interspecific genetic variation of the genus *Populus* have focused on a limited number of markers, like AFLPs and microsatellites, distributed along the genome (Cervera et al. 2001; Cervera et al. 2005; Fossati et al. 2005; Rohde et al. 2011). With the aim of characterizing the poplar pan-genome and investigating its relationship with the origin of intra- and interspecific diversity, we performed a genome-wide analysis of structural variation between three poplar species: *Populus nigra, Populus deltoides*, and *P. trichocarpa*. We obtained a sequencing depth ranging from approximately 26× to 45× in four *P. nigra* genotypes (*BDG*, *71077-308*, *BEN3*, and *Poli*), two *P. deltoides* accessions (*L150-089* and *L155-079*) and in the *P. trichocarpa* genotype used to build the reference genome (*Nisqually-1*). These high-depth samples underwent deep structural variation analysis. The 15 low-depth *P. nigra* accessions were pooled and analyzed together with the aim of studying the incidence of SV in a *P. nigra* discovery panel. Sequencing depth and library statistics for each of the resequenced accession are reported in supplementary tables S1 and S2 (Supplementary Material online). In the high-depth *P. nigra* and *P. deltoides* samples, we covered about 82% of the *P. trichocarpa* reference genome considering all the aligned reads and about 63% of the reference genome considering only the reads that were uniquely aligned to the genome. These results showed the presence of a high degree of genome similarity between the three poplar species included in the study. As expected, in the *P. trichocarpa Nisqually-1* sample, we covered a higher fraction of the genome corresponding to 97% when considering all the aligned reads and 92% when considering only the reads uniquely aligned to the reference.

### INDELs Affect a Large Portion of Poplar Genome and Are Frequently Related to LTR-Retrotransposons

*P. nigra* and *P. deltoides* belong to the same section of the genus *Populus* (Stettler et al. 1996; Cervera et al. 2005) and are phylogenetically more closely related to each other than they are to *P. trichocarpa* (see table 1 for a schematic representation of the known phylogenies). Thus, using the *P. trichocarpa* v3.0 genome as a reference, we expected to detect in *P. nigra* and *P. deltoides* INDELs that occurred after the divergence of the two species from *P. trichocarpa*. In particular, INDELs that occurred before the *P. nigra*/*P. deltoides* speciation (at time *c* in the phylogenetic tree in table 1) are expected to be shared by the two species, while more recent ones (times *d* and *e*) are expected to be species specific. In *P. trichocarpa*, a different scenario is expected. In fact, the *P. trichocarpa* reference genome is a combination of the two haplotypes of the highly heterozygous individual *Nisqually-1*, which is the same individual we had included in the study. Thus, in *P. trichocarpa*, we expected to identify only variants that are heterozygous in *Nisqually-1* and, thus, not fixed in this species.

**Table 1:** Summary Statistics of INDELs Detected in the High-depth Poplar Accessions.

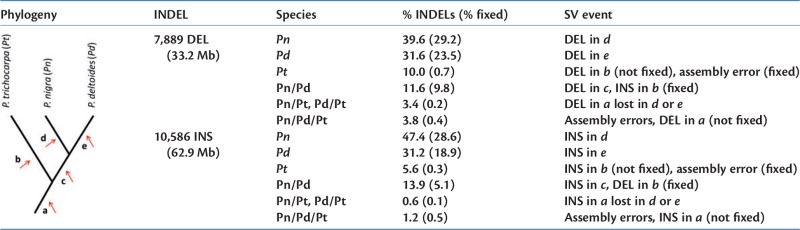

Note.—The table reports a schematic representation of the phylogeny of the three studied poplar species and a classification of the detected deletions (DEL) and insertions (INS) on the bases of the species in which variants have been detected: *Pn, Pd, Pt *=* P. nigra, P. deltoides*, and *P. trichocarpa*-specific INDELs, respectively; *Pn*/*Pd*, *Pn*/*Pt*, *Pd*/*Pt *=* *INDELs detected in the pairs *P. nigra–P. deltoides, P. nigra–P. trichocarpa*, and *P. deltoides–P. trichocarpa*, respectively; *Pn*/*Pd*/Pt = INDELs detected in all the three studied species. For each category, the table reports the percentage of detected variants (% INDELs) belonging to that category, the percentage of variants detected in homozygous state in all individuals (%fixed) and the structural variation event (SV event) responsible for the correspondent INDEL in agreement with the known phylogeny and under the assumptions of all mutations having occurred only once and of absence of false positives and false negatives.

In total, we detected 7,889 deletions and 10,586 insertions relative to the *P. trichocarpa* reference genome, covering, respectively, 33.2 Mb and 62.9 Mb of genomic sequence. The median length of detected variants was 2,176 bp and 4,860 bp, respectively. The number of detected variants and the number of megabases involved in each studied sample are reported in supplementary table S4 (Supplementary Material online), while the complete lists of insertions and deletions are reported in supplementary tables S5 and S6 (Supplementary Material online), respectively. Using a PCR-based assay, we experimentally validated a randomly selected set of 29 INDELs: 14 out of 16 tested insertions (supplementary fig. S3, Supplementary Material online) and 13 out of 13 tested deletions (supplementary fig. S4, Supplementary Material online) were confirmed by PCR (see Section 5 of supplementary material, Supplementary Material online, for details). The validation of almost 2,400 deletions of the sample *Poli* was also performed *in silico* using a *P. nigra de novo* draft assembly obtained with the short reads of *Poli* (see Section 6 of supplementary material, Supplementary Material online, for details). About 98.4% of the tested deletions were confirmed by this analysis, suggesting a high accuracy of the detection process. Due to major difficulties in the *de novo* assembly process of genomic regions containing the insertions, we could not replicate this analysis to test the accuracy of the detection of insertions (see Section 6 of supplementary material, Supplementary Material online, for details).

In plants, transposable elements are a major source of genetic variation (Kidwell and Lisch 1997). As expected, a great proportion of the detected deletions (55.7%) were highly homologous to known TEs and, as required by the applied detection pipeline, almost all the detected insertions were related to the presence of transposable elements (table 2). In particular, 61.8% of the classified deletions and 83.8% of the classified insertions resulted to be homologous to class I retroelements while the remaining were classified as class II DNA TEs. LTR Gypsy elements were the most represented among class I elements covering 39.9% and 49.8% of the classified deletions and insertions, respectively. Among class II elements, the most represented were hAT elements, followed by Helitrons and CACTA elements. The percentage of deletions not classified as TEs was lower in the dataset of variants shared by *P. nigra* and *P. deltoides* species with respect to species specific ones (supplementary table S7, Supplementary Material online). These observations are in accordance with the model proposed in table 1 and with the known molecular mechanisms that can generate SV of different origin: class I elements move strictly through a copy and paste mechanism and should always generate insertions, class II elements usually move through a cut and paste mechanism and should produce both insertions and deletions, the non-annotated deletion events are unrelated to transposition activity and should represent real deletions as a consequence of defective repair events following double-strand breaks. The increased relative proportion of class II elements in comparison with class I observed in deletions and especially in those that are only identified in *P. nigra* or *P. deltoides* samples is, therefore, a strict consequence of their different transposition mechanism. As expected, LTR retrotransposons involved in deletions resulted to be younger than those fixed in the studied poplar species (supplementary fig.S5). However, difference in age distributions was not as pronounced as that observed in cultivated plants such as maize (Brunner et al. 2005), rice (Hurwitz et al. 2010), or grapevine (Michele Morgante, personal communication). This difference could be explained by the presence in the studied poplar species of a lower rate of recent activity of TEs, a higher level of incomplete lineage sorting or/and a higher level of gene flow. We observed, in insertions shared by *P. nigra* and *P. deltoides* samples, a significant increase of annotated class I retroelements mainly due to a higher proportion of gypsy elements (supplementary table S8, Supplementary Material online) suggesting that the insertion events of gypsy elements are in general older than copia ones. LTR retrotransposons vary in size from several hundred bases to over 10 kb (Bennetzen 2000). Plotting the size distribution of the identified INDELs (supplementary fig. S6, Supplementary Material online), we observed three signature peaks at 8.5–9.5 kb, 4–5 kb, and at 1–2 kb that could be, respectively, related to entire gyspsy LTR-retrotransposons, copia ones, and to retroelements that underwent subsequent rearrangements.

**Table 2:** Classification of INDELs on the Basis of their Homology with Class I (retrotransposons) or Class II (DNA transposons) Transposable Elements

		# DEL^a^	% DEL^b^ (%)	# INS^c^	%INS^d^
Class I	LTR Gypsy	1,752	39.9	5,175	49.8
	LTR Copia	854	19.4	2,862	27.5
	LINE L1	64	1.5	365	3.5
	LTR	38	0.9	308	3.0
	SINE	6	0.1	2	0.0
	Total	2,714	61.8	8,712	83.8
Class II	TIR hAT	525	12.0	449	4.3
	Helitron	380	8.7	255	2.5
	TIR CACTA	343	7.8	481	4.6
	TIR PIF/Harbinger	39	0.9	93	0.9
	TIR Mutator	26	0.6	0	0.0
	Class II unknown	365	8.3	410	3.9
	Total	1,678	38.2	1,688	16.2
Unclassified		3,497	44.3	186	1.8

^a^Number of classified deletions.

^b^Percentage of classified deletions.

^c^Number of classified insertions.

^d^Percentage of classified insertions.

The majority of variants were detected only in one of the three analyzed species (table 1), suggesting that INDELs are mainly species-specific and in agreement with a recent origin following separation of species (times *b*, *d*, or *e* in table 1). As expected, the majority of *P. trichocarpa*-specific variants were not fixed in the species and represent heterozygous INDELs of the sample *Nisqually-1*. The small fraction of homozygous INDELs detected in *Nisqually-1* may represent false-positive variants, errors in the assignment of the genotype or inaccuracies in the reference genome assembly. For deletions, the latter case was supported by the observation that an important fraction of the homozygous deletions detected in *Nisqually-1* contained a stretch of 100 or more “N” bases in the corresponding region of the reference genome, denoting possible inaccuracies in the scaffolding step of the assembly process.

On the contrary, a great proportion of both *P. nigra* and *P. deltoides*-specific variants were fixed in the species (table 1). This was confirmed by the analysis performed in the low-depth *P. nigra* pool in which we found that for both types of variants, the majority of INDELs (78.2% of deletions and 61.7% of insertions), had a frequency higher than 0.9 suggesting that a great proportion of variants are fixed in this species (supplementary fig. S7, Supplementary Material online).

INDELs shared between *P. nigra* and *P. deltoides* species represent 11.6% and 13.9% of the total dataset of deletions and insertions, respectively (table 1). A high proportion of variants shared between this two species are consistent with the known phylogeny of the three studied species. In particular, on one hand, deletions shared by *P. nigra* and *P. deltoides* samples may represent real deletion events that occurred before the *P. nigra*/*P. deltoides* speciation (time *c*) or *P. trichocarpa*-specific insertion events (occurred at time *b*). On the other hand, insertions shared by this two species may result from insertion events that occurred before speciation (time *c*) or deletion events that occurred in *P. trichocarpa* (time *b*).

A very small fraction of INDELs (3.4% of deletions and 0.6% of insertions) resulted to be shared between either *P. nigra* or *P. deltoides* samples and *P. trichocarpa*. This scenario can be the result of old deletion or insertion events occurred in the common ancestor (time *a*) that have been lost in *P. nigra* or *P. deltoides* at a later stage. Finally, 3.8% of deletions and 1.2% of insertions were detected in all the three species. These variants may arise from inaccuracies in the reference genome or, in the case of not fixed variants, may represent old deletion or insertion events (time *a*).

**Fig. 1:**
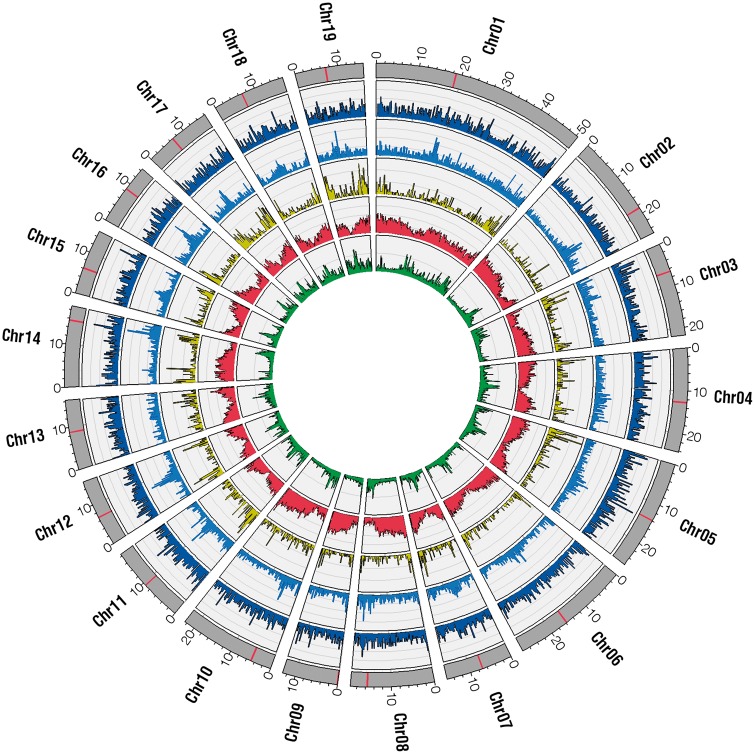
Genomic distribution of INDELs and genic CNVs. The number of deletions (dark blue track, *y* axes range = 0–20), insertions (blue track, *y* axes range = 0–40), genic CNVs (yellow track, *y* axes range = 0–15), annotated genes (red track, *y* axes range = 0–80), and the repetitiveness of the genome (green track, *y* axes range = 0–10) are represented in windows of 250 kb along the 19 *Populus trichocarpa* chromosomes (outer gray bars). Predicted centromeric regions are highlighted in red.

In plants, the centromeric and pericentromeric chromosome regions are colonized by LTR-gypsy retroelements, which are the most abundant repetitive elements in the *P. trichocarpa* genome (Jiang et al. 2003; Wang et al. 2008). By plotting the distribution of the detected INDELs along the 19 poplar chromosomes, we noticed that deletions were evenly distributed through the genome, showing only a slight preference for highly repeated regions while insertions were preferentially located in centromeric regions (fig. 1). On one hand, significant enrichment of deletions in the centromeric regions was observed only in Chr14 and Chr16 (*P* < 0.05 assuming a Poisson distribution for the null hypothesis). On the other hand, significant enrichment of insertions in the centromeric region was observed in chromosomes 1, 2, 3, 8, 9, 10, 11, 12, 14, 15, 16, 17, 18, and 19 (*P* < 0.05 assuming a Poisson distribution for the null hypothesis).

**Fig. 2:**
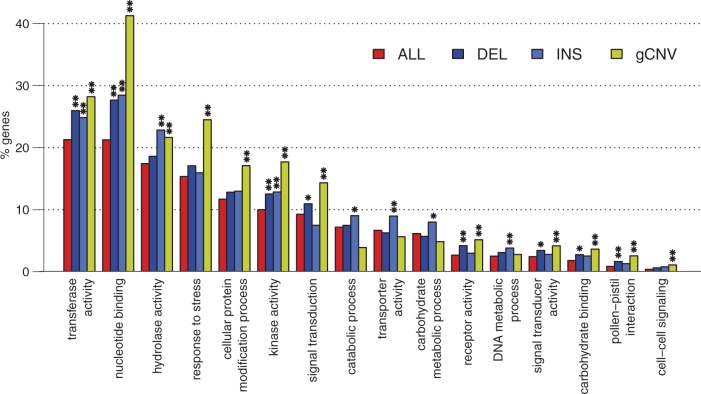
Frequencies of the Gene Ontology terms for which an over-representation has been observed when comparing the subsets of genes included in deletions (DEL), genes interrupted by insertions (INS) and genic CNVs (gCNV) in the studied samples with respect to the complete dataset of *P. trichocarpa* annotated genes (ALL). * *P*-value <0.05, ** *P*-value < 0.01.

We investigated the relationship between the number of detected INDELs in a given region of the genome and its repetitiveness: 250 kb long genomic regions carrying six or more insertions showed a significantly higher repetitiveness compared with those not carrying insertions (supplementary fig. S8, left panel, Supplementary Material online). The same was true for regions carrying seven or more deletions (supplementary fig. S8, right panel, Supplementary Material online). This suggests that both deletions and insertions are preferentially located in highly repetitive regions. According to the *P. trichocarpa* v3.0 gene annotation, the deleted sequences contained a total of 2,928 annotated genes while 1,986 annotated genes were interrupted by a predicted insertion. Seventy-one percent of the genes affected by deletions and 78% of those affected by insertions were associated to at least one Gene Ontology term. We detected eight GO categories over-represented in deleted genes and eight GO categories over-represented in genes interrupted by insertions (fig. 2). Three of them (“transferase activity”, “nucleotide binding”, and “kinase activity”) were over-represented in both types of variants. The gene content analysis of the deleted sequences confirmed that a great part of the deletions resulted from the activity of transposable elements and suggested that insertions have preferentially occurred in regions rich in transposable elements. In fact, the functional annotation showed a significant enrichment of genes with GO molecular functions (transferase activity, nucleotide binding, and hydrolase activity) that can be related to TE proteins, like the Gag-Pol polyprotein (Feschotte et al. 2002).

### Genic CNVs Preferentially Affect Genes Located near Telomeres and Related to Defense Traits

To get a more comprehensive insight into the effects of structural variation on genes, we detected genic CNVs using the depth of coverage signature of NGS. We detected 3,230 genes showing copy number variation between at least two of the five high-depth samples (supplementary table S9, Supplementary Material online). A total of 989 genic CNVs overlapped with deletions (647), insertions (212) or both kinds of INDELs (130). The distance between genic CNVs and INDELs was significantly shorter than that between all the annotated genes and INDELs, suggesting that genic CNVs tend to occur near transposable elements (supplementary fig. S9, Supplementary Material online). The number of genic CNVs detected in each pairwise comparison is reported in supplementary table S10 (Supplementary Material online). We observed that genic CNVs are often organized in clusters (*P* = 0.0003), and that windows containing clusters of genic CNVs have a higher gene density (see supplementary fig. S10, Supplementary Material online). Genic CNVs are not uniformly distributed along the poplar genome (fig. 1): the number of genic CNVs is significantly higher than expected within three Mb from chromosomes telomeres and significantly lower than expected at distances greater than 7 Mb from the telomere (supplementary fig. S11, Supplementary Material online). These findings are in agreement with previous reports on an over-representation of CNVs in the subtelomeric regions of the human genome (Nguyen et al. 2006; Riethman 2008). Similar to what we previously observed for INDELs, the average number of genic CNVs detected by comparing two samples belonging to a different species (1,147) was greater than the average number of genic CNV detected by comparing samples of the same species (747). A total of 404 genic CNVs were *P. nigra* (230) or *P. deltoides* (174) intraspecific variations, 367 were specifically detected by comparing *P. nigra* with *P. deltoides* samples, 187 by comparing the *P. nigra* samples with the *P. trichocarpa* one and 213 by comparing *P. deltoides* and *P. trichocarpa* samples. Inter- and intraspecific genic CNVs are distributed along the whole genome (supplementary fig. S12, Supplementary Material online) but it is possible to observe some genomic locations enriched for some inter- or intraspecies-specific variants. For example, the last 2 Mb of Chr07 are enriched for *P. nigra* intraspecific genic CNVs, the region comprised between 46 and 48 Mb of Chr01 is enriched for *P. deltoides* intraspecific ones while the region between 4 and 6 Mb of Chr14 is enriched for genic CNVs detected by comparing *P. nigra* with *P. deltoides* samples. *P* values calculated across the whole genome are reported in supplementary table S11 (Supplementary Material online).

Among the 3,076 (95.2% of the total) genic CNVs having a BLASTx hit against the *Viridiplantae* section of the RefSeq protein database, 2,252 (∼70%) were associated to at least one Gene Ontology term. We detected 12 GO categories significantly over-represented with respect to the whole set of *P. trichocarpa* genes (fig. 2). Three of them (“transferase activity”, “nucleotide binding”, and “kinase activity”) were over-represented also in genes affected by both deletions and insertions, five (“signal transduction”, “receptor activity”, “signal transducer activity”, “carbohydrate binding”, and “pollen-pistil interaction”) were over-represented also in genes affected by deletions and one (“hydrolase activity”) was over-represented also in genes affected by insertions. Transposable elements make up a high proportion of repetitive DNA, indicating a possible contribution of these elements to the formation of the genic CNVs localized in highly repetitive genomic regions. In fact, the functional annotation showed a significant enrichment in the dataset of genic CNVs associated with GO molecular functions (“transferase activity”, “nucleotide binding”, and “hydrolase activity”) that can be related to TE proteins. LTR retroelements, representing the most active class of transposable elements in poplar, have been previously shown to be major contributors to genome size evolution in rice (Ma and Bennetzen 2004). Transposable elements may also be important in new gene formation and genome evolution. For example, *Helitron*-related transposable elements have been shown to carry pseudogenes in maize and to contribute to the expansion and evolution of the maize genome (Morgante et al. 2005; Yang and Bennetzen 2009). Genic CNVs enriched for the 12 GO categories are not uniformly distributed through the genome and the enrichment for genic CNVs of a given GO category is not necessarily due to local enrichment of genes belonging to the same GO category (supplementary fig. S13, Supplementary Material online). *P* values calculated across the whole genome are reported in supplementary table S12 (Supplementary Material online). For example, in the terminal part of Chr01 (approximately between 45 and 48 Mb), there is an enrichment for genic CNVs in genes belonging to the three GO categories “nucleotide binding”, “hydrolase activity”, and “response to stress”, whereas the number of genes belonging to these categories was not significantly different from expectations; these three categories are all related to disease resistance and in fact the presence of NBS-LRR genes has been previously reported in this genomic location (Kohler et al. 2008).

Approximately 16% of genic CNVs encode for disease resistance proteins, such as the Nucleotide Binding Site-Leucine Rich Repeat (NB-LRR) gene family. In maize, evidences that disease resistance genes exhibit copy number variation for different haplotypes have already been reported (Smith et al. 2004). In addition, the overrepresentation of disease resistance genes in regions of structural variation between different species has already been reported for rice (Hurwitz et al. 2010). Evidences of the high variability of disease resistance genes have been also reported for *Arabidopsis thaliana* (Clark et al. 2007). In *A. thaliana*, tandem duplications and losses have been found to play the dominant role in affecting copy number of disease resistance genes (Cannon et al. 2004). Tandem duplication processes are considered to be a major cause for the generation of clusters of duplicated genes and for the expansion of some gene families. For example, the extant distribution and diversity in *Arabidopsis* genome of the NBS-LRR sequences has been generated by extensive duplication and ectopic rearrangements that involved segmental duplications (Meyers et al. 2003). Unequal recombination occurring when interspersed repetitive elements promote non-homologue crossing-over is thought to be the primary mechanism driving the expansion of gene clusters (Leister 2004). After the duplication, each paralogous gene may retain the same function as the ancestral copy or may lose the original function and/or obtain a new function. Genes that confer a selective advantage are thus maintained by natural selection. Therefore, the genes that we have found to be over-represented in the genic CNVs may reflect recent gene acquisition or gene loss events occurred in one of the two poplar species and are candidate markers of interspecific divergence.

### INDELs and CNVs Disrupt Gene Structure and Affect Gene Function in Lowly Expressed and Rapidly Diverging Genes

A total of 28.6% of the deletions contained one or more annotated genes while 20.4% of the insertion sites were predicted to overlap an annotated gene. The number of genes affected by deletions (2,928) and insertions (1,986) was significantly lower than expected by chance (4,240 and 2,716 respectively, confidence intervals: 4,168–4,335 and 2,634–2,828). Moreover, 16.6% (1,310) of the deletions and 10.4% (1,101) of the insertions were located at less than 500 bp from an annotated gene, against expectations of 1,437 (confidence interval 1,377–1,501) and 1,923 (confidence interval 1,848–2,001), respectively, based on simulations of randomly distributed deletion and insertion points in the genome. Approximately 43% of the genes affected by deletions were completely deleted, while the remaining genes were only partially deleted. In total, 0.09 Mb of 5′ UTR regions, 0.19 Mb of 3′ UTR regions, 1.76 Mb of coding regions, and 2.37 Mb of intronic regions resulted to be deleted in at least one sample. The majority of the insertions affecting a gene were observed in intronic regions (807), followed by those observed in coding regions (575), in 3′ UTR regions (218) and in 5′ UTR regions (75); the remaining sites were not univocally detected in a specific genic region. The extent of each genic portion affected by deletions was shorter than expected by chance, while for those affected by insertions this was observed only in CDS and introns (supplementary table S13, Supplementary Material online).

**Fig. 3:**
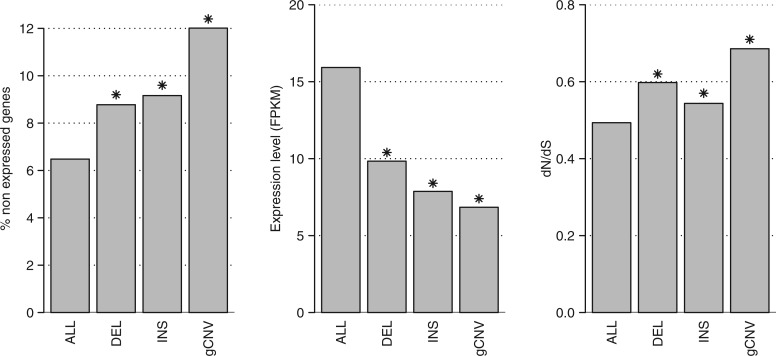
Genetic features associated with *P. trichocarpa* transcriptome (ALL), genes disrupted by deletions (DEL), genes disrupted by insertions (INS), and genes affected by genic CNVs (gCNV). *Left panel*: percentage of non-expressed genes. The percentage of non-expressed genes was significantly higher in genes affected by any SV (deletion, insertion or genic CNV) than the whole transcriptome. *Central panel*: expression levels in the whole transcriptome and in genes affected by SV. *Right panel*: rates of nonsynonymous to synonymous changes in whole transcriptome and in genes affected by SV. d*N*/d*S* values in genes affected by SV were significantly higher compared with whole transcriptome. *P-value < 0.05.

Our results showed that INDELs tend to occur outside of genic sequences, probably due to the effect of purifying selection. When SV affect genes, they are more likely to affect genes that are not expressed or that show low levels of expression (fig. 3, left panel). In fact, if compared with the whole dataset of *P. trichocarpa*-annotated genes, both genic CNVs and INDELs resulted to affect a higher proportion of genes for which no expression has been detected by analyzing the *P. trichocarpa* RNAseq data obtained from four different tissues. In the complete dataset, the percentage of non-expressed genes was 6.5%, while this value significantly increased in genes interrupted by insertions (9.2%, chi-square test *P*-value 1.2E−06), in deleted genes (8.8%, chi-square test *P*-value 4.5E−07) and in genic CNVs (12.0%, chi-square test *P*-value 2.5E−37) (fig. 3, left panel). We repeated the same analysis separately for genes affected by INDELs detected in homozygous state in at least one of the studied samples and for those detected only in heterozygous state. This analysis showed that the significant increase of non-expressed genes is driven by the 2,270 and 1,346 genes affected by homozygous deletions and insertions respectively (supplementary fig. S14, Supplementary Material online). This marked difference between homozygous and heterozygous structural variants could indicate the presence of a fraction of variants with potential deleterious effects on expression that are only found in heterozygous condition and could contribute to the genetic segregational load of the poplar species considered. We compared expression levels of all expressed genes with those of genes affected by insertions, deletions or CNVs. We found that expression levels of genes affected by structural variation are significantly lower if compared with the expression levels of all the expressed genes (fig. 3, central panel). Analyzing the four tissues (developing xylem, leaf, callus from root, and cambium) separately, we observed the same pattern. Genes affected by SV showed significantly reduced levels of expression in the four studied tissues with the exception of callus, in which the reduction was not significant in genes affected by deletions (supplementary fig. S15, Supplementary Material online).

To determine whether genes affected by structural variation were generally associated with fast evolving proteins, we examined the rates of non-synonymous to synonymous changes per gene (d*N*/d*S*). Using a randomization test, we found that genes affected by all the three types of studied structural variation had a significantly higher d*N*/d*S* ratio compared with non-affected genes (fig. 3, right panel). In line with what was recently reported for *Arabidopsis thaliana* (Bush et al. 2014), these results suggest the existence of either positive selection or relaxed negative selection in the genes affected by structural variation. Part of the genes affected by structural variation may represent pseudogenes or wrong predictions in the genome annotation process. On one hand, the increased d*N*/d*S* values in genes affected by both deletions and insertions are mainly due to the significant higher d*N*/d*S* values of genes in which the INDEL specifically disrupt a CDS (supplementary fig. S16, Supplementary Material online). On the other hand, d*N*/d*S* values of genes in which deletions affected only the intronic regions or in which insertions occurred in 3′ UTRs are significantly lower (supplementary fig. S16, Supplementary Material online).

### Identification of INDELs and Genic CNVs Allows Reconstructing the Poplar Pan-Genome

This study demonstrated that structural variation contributes to a substantial amount of the overall genetic variation in poplar. The detected deletions and insertions cover approximately 20% of the poplar reference genome. We found that a great proportion of this variability was driven by two main mechanisms: the activity of transposable elements (especially class I LTR retroelements) and non-homologous recombination driven by repeated gene clusters. A predominant contribution of transposable elements to the genome structural variation was already reported for maize (Morgante et al. 2007).

**Fig. 4:**
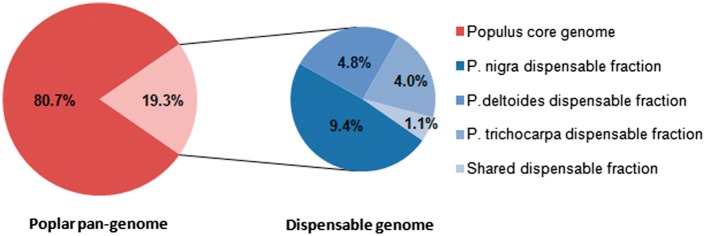
Composition of poplar pan-genome constructed using the structural variants detected in the seven high-depth accessions.

By summing the 434 Mb included in the *P. trichocarpa* reference genome and the 63 Mb of sequence detected as insertions, we estimate a poplar pan-genome size of approximately 497 Mb. According to our results, 80.7% (401 Mb) of the poplar pan-genome is shared by all the seven studied accessions and constitutes the “core” genome (fig. 4). The remaining portion of the pan-genome (19.3%, 96 Mb) is present in more than one, but not all the seven poplar accessions, and represents the “dispensable” genome. We estimate the contribution of each of the three studied species to the dispensable genome: for the most part, the dispensable genome is made up by INDELs detected only in *P. nigra* accessions (9.4% of the pan-genome); while *P. deltoides* and *P. trichocarpa*-specific INDELs originate a smaller fraction of the dispensable genome (4.8% and 4.0% of the pan-genome, respectively). This difference is probably attributable to the higher number of studied *P. nigra* samples. The fraction of dispensable genome made up by INDELs shared by different species is very small (1.1% of the pan-genome), indicating that the majority of the poplar dispensable genome is made up by species-specific variation. Estimated poplar core and dispensable genome fractions are very similar to those reported for soybean that were obtained by analyzing seven assembled *Glycine soja* genomes (core: 80.1%, dispensable: 19.9%; Li et al. 2014). On the contrary, a significantly higher fraction of dispensable genome has been reported in maize where a comparison across four randomly chosen genomic regions between two inbred lines showed that only 50% of the sequences were shared by the two genotypes (Morgante et al. 2007). Gypsy and Copia LTR-retrotransposon insertions in the *P. trichocarpa* genome are older than those observed in maize, with a much larger fraction of elements that are older than 5 million years (Cossu et al. 2011). This difference in recent transposition activity could partly account for the observed differences between the two species.

We observed that the dispensable fraction of the poplar pan genome is mainly composed by repeated sequences such as those typical of transposable elements. However, we found that 2,270 genes (5.5% of the annotated ones) were deleted in homozygous state in at least one accession and thus belong to the dispensable fraction of the genome. In addition, 2,453 genes (5.9% of the annotated ones) have been detected as genic CNV and thus are present with a variable copy number in the studied samples. The detection of INDELs in *P. nigra* low-depth pool showed that a great proportion of variants are fixed in the species (supplementary fig. S7, Supplementary Material online). However, using results obtained for the four high-depth *P. nigra* accessions we observed that each individual contributed to the total number of INDELs with private variants (supplementary fig. S17, Supplementary Material online). This observation suggests that increasing the number of studied individuals would allow the identification of additional portions of the dispensable genome of the genus *Populus*. Moreover, in this study, we have used the genome of *P. trichocarpa* as a reference to detect INDELs and thus we did not sample the portion of the dispensable genome made up by structural variation specific to the *P. nigra* or *P. deltoides* genomes that does not correspond to TE insertions.

Studies focused on the detection and characterization of structural variation at a genome-wide scale in plants, and in particular in tree species, are still in their infancy. In the last years, few studies have been carried out in different plant species such as maize (Lai et al. 2010; Chia et al. 2012), rice (Xu et al. 2012), Arabidopsis (Lu et al. 2012), sorghum (Zheng et al. 2011), soybean (Lam et al. 2010; Li et al. 2014), and melon (Sanseverino et al. 2015). In this study, we performed a genome-wide comparative analysis of three closely related *Populus* species, we provided a first detailed catalogue of structural variants across the whole genome and we proposed a functional and structural characterization of poplar pan-genome. Future functional studies of the detected variants could help understanding the mechanisms of speciation in poplar as well as the role of artificial and natural selection in these genomes. Understanding the role of inter- and intraspecific structural variants in poplar phenotype may have important implications for breeding, particularly, interspecific hybrids.

## Materials and Methods

### Study Sample

Two naturally occurring *Populus deltoides*, 1 *P. trichocarpa* and 19 *P. nigra* individual trees were analyzed in the study (the complete list is reported in supplementary tables S1 and S2, Supplementary Material online). Plant material for DNA sequencing of *P. deltoides* samples was obtained from the Unité Amélioration Génétique et Physiologie Forestières—I.N.R.A. (Orléans, France). Illumina DNA sequences of the *P. nigra* genotypes were obtained as previously described (Faivre-Rampant et al. 2016). Illumina DNA sequences of the *P. trichocarpa* sample *Nisqually-1* and *P. trichocarpa* RNAseq data from four different tissues (developing xylem, leaf, callus from root, and cambium) were retrieved from the NCBI Sequence Read Archive (run accessions are reported in supplementary tables S1 and S3, Supplementary Material online, respectively).

### DNA Extraction, Library Preparation, and Sequencing

Leaf tissues from greenhouse or field-grown plants were ground in liquid nitrogen and high-molecular-weight genomic DNA was extracted from nuclei as previously described (Zhang et al. 1995). The protocol was improved with the addition of PVP40 both in the wash (5%) and the lysis (2%) buffers. To prepare 2×100 bp paired-end libraries, 5 µg of nuclear DNA were randomly fragmented by Fragmentase treatment (NEBNext™ dsDNA Fragmentase™, New England Biolabs) at 37 °C for 1 h. Libraries were prepared using Illumina reagents, according to manufacturer's specifications (Illumina, San Diego, CA). End repair of fragmented DNA was performed using T4 DNA polymerase and Klenow polymerase with T4 polynucleotide kinase. Subsequently, an “A” base was added at the 3' end using a 3'–5' exonuclease-deficient Klenow fragment. The paired-end adaptor with a single T base overhang at the 3′ end was ligated to the above products. The PE adaptor ligated products were separated on a 2% agarose and the 400–600 bp size fraction was excised from the gel. Fragments were enriched by 16-cycle PCR reaction using PE primers 1.1 and 2.1 (Illumina, San Diego, CA). Whole-genome re-sequencing was performed at the Institute of Applied Genomics (IGA, Udine, Italy) facilities using either a GAII analyzer or Hiseq 2000 platforms from Illumina (Inc. San Diego, CA, USA). Images from the instruments were processed using the manufacturer’s pipeline software to generate FASTQ sequence files.

### Sequencing Data Analysis

Adaptor sequences and low-quality 3' ends were removed from both DNA and RNA short reads using, respectively, cutadapt (Martin 2011) and ERNE-FILTER (http://erne.sourceforge.net) with default parameters. To assess the quality of sequencing data, Jellyfish (Marçais and Kingsford 2011) was used to obtain the kmer spectra plots with a *k* value of 16. After trimming, pairs with both reads longer than 50 bp were aligned to the v3.0 of the *P. trichocarpa* reference genome (Tuskan et al. 2006), retrieved from the JGI Comparative Plant Genomics Portal (www.phytozome.net/poplar.php, last accessed August 5, 2016). Alignment was performed using the short read aligner BWA (Li and Durbin 2009) with default parameters. After alignment, duplicated sequences were removed using the samtools *rmdup* utility (Li et al. 2009). The mean sequencing depth for each individual was calculated dividing the total number of uniquely aligned bases by the number of covered positions. Seven samples (four *P. nigra*, two *P. deltoides*, and one *P. trichocarpa* individuals) were sequenced at higher depth and underwent extensive investigation; they will be referred to as “high-depth” in the subsequent sections of the paper. The remaining 15 *P. nigra* samples were sequenced at a variable lower depth and were analyzed together as a pool; they will be referred to as “low-depth” in the subsequent sections of the paper. To obtain a pool in which each sample was equally represented, we reduced the depth of the more covered samples to an approximate sequencing depth of 5× (which is the sequencing depth of the less covered samples). The number of reads selected for each sample and used to create the pool is reported in supplementary table S2 (Supplementary Material online).

RNAseq data was aligned using TopHat2 (Kim et al. 2013) by requiring a minimum segment length of 25 bp (i.e., the minimum length of the independently mapped segments generated by splitting a read) and allowing not more than one mismatch per mapped segment. Alignments of RNAseq reads were analyzed with the tool *htseq-count* included in the package HTSeq v0.6.1 (Anders et al. 2014) to count the number of sequenced fragments aligned in each of the 41,335 genes included in the *P. trichocarpa* v3 annotation gff file.

### Structural Variants Analysis

Two different classes of structural variants were investigated: (1) insertions/deletions (INDELs) related with the activity of transposable elements and (2) genic copy number variants (genic CNVs). The detection of INDELs was performed at individual level in high-depth individuals, while the 15 low-depth samples were pooled and analyzed together in order to increase the understanding of the contribution of structural variation to *P. nigra* genetic diversity. INDELs were classified in species-specific variants (i.e. variants detected only in samples of the same species) and variants shared by species (i.e. variants detected in samples belonging to different poplar species). The study sample included a different number of *P. nigra* and *P. deltoides* samples (four *P. nigra* and two *P. deltoides*); to obtain a more realistic estimation of the contribution of each of the two species to poplar structural variation, this classification was performed by using all the possible combinations of only two *P. nigra* accessions and averaging the results. The detection of copy number variations affecting genes was performed by pairwise comparisons between five high-depth samples: two *P. nigra* (*Poli* and *71077-308*), the two *P. deltoides* (*L150-089* and *L155-079*) and the *P. trichocarpa* sample (*Nisqually-1*). The remaining two high-depth *P. nigra* samples (*BDG* and *BEN3*) were not employed for this analysis due to their altered kmer profiles (supplementary fig. S1, Supplementary Material online) denoting possible bias in library complexity that could lead to loss of power in the depth of coverage-based analysis.

### Detection of Deletions

To evaluate the performance of different available methods in the detection of deletions and to choose the best performing ones on our data, a simulation experiment was conducted by simulating 1,000 deletions and insertions in the *P. trichocarpa* reference genome (see Sections 1–3 of supplementary materials, Supplementary Material online, for details). According to simulation results, the detection of deletions with respect to the *P. trichocarpa* reference genome was performed by combining the results of the two SV detection methods DELLY (Rausch et al. 2012) and GASV (Sindi et al. 2009).

DELLY was run with default parameters and the results were filtered by selecting only the deletions having a length included in the range 1–25 kb and by discarding those supported by less than five paired-ends or by reads with a median mapping quality lower than 20. GASV was run with default parameters and the results were filtered by selecting only the deletions having a length included in the range 1–25 kb and being supported by at least five paired-ends. Since GASV reports two intervals in which the left and the right breakpoints of the predicted deletions are supposed to be included, the central points of the intervals were used to approximate the two breakpoints of each deletion. Predictions obtained by the two tools for each sample were merged using a confidence interval of 250 bp around the breakpoints: deletions with overlapping confidence intervals at both sides were combined into a single event. Simulation results showed that DELLY is more accurate than GASV in the estimation of the breakpoints. Thus, the breakpoints estimated by DELLY were assigned to deletions detected by both tools. Deletions detected in the seven high-depth individuals and in the low-depth pool were merged using a confidence interval of 500 bp around the breakpoints. The integrated list of deletions was analyzed with a custom python script (see Section 2 of supplementary materials, Supplementary Material online, for details) to calculate the proportion of reads supporting the deletions in each sample and thus assign the genotype (non-carrier, heterozygous carrier, and homozygous carrier), based on the individual allele frequency of the variant. The genotype was assigned only to samples having a minimum sequencing depth of 5 in correspondence to the deletion breakpoints. The individual allele frequency (i.e. the proportion of reads carrying the variant) was employed to refine deletion calls in all individuals and to assign the genotype: individuals in which the alternative allele had a frequency lower than 0.2 were classified as non-carriers; individuals carrying variants with a frequency included in the range 0.2–0.8 were considered heterozygous carriers and those carrying variants with a frequency higher than 0.8 were considered homozygous carriers.

### Annotation of Deletions

The sequences of the deletions were extracted from the *P. trichocarpa* v3.0 reference genome and annotated to identify their homology with known plant transposable elements. The annotation process consisted of three consecutive approaches:

First, the coordinates of the deletions were interpolated with the *P. trichocarpa* v3.0 repeats annotation file (Ptrichocarpa_210_v3.0.repeatmasked_assembly_v3.0.gff3) retrieved from the poplar section of the Joint Genome Institute website (http://phytozome.jgi.doe.gov/pz/portal.html#!bulk?org = Org_Ptrichocarpa) to identify deletions overlapping with already annotated transposons.

Deletions not annotated by the previous step were analyzed with the tool *TEannot* included in the REPET package v2.2 (Flutre et al. 2011), which annotates transposable elements (TEs) in genomic sequences using a library of known TE sequences. *TEannot* was run with default parameters and using RepBase18.09 (Jurka et al. 2005) as a reference database.

Finally, the remaining deletions were aligned against a database of transposable elements with BLASTn (Altschul et al. 1990) using an *E*-value threshold of 10^−20^. The database consisted of the plant section of RepBase18.09 database (Jurka et al. 2005), a list of *P. trichocarpa* LTR retrotransposons retrieved from http://www.agr.unipi.it/ricerca/plant-genetics-and-genomics-lab/sequence-repository (last accessed August 5, 2016) and a set of poplar repeats *de novo* detected and classified by analyzing the *P. trichocarpa* reference genome with *RepeatModeler* (http://www.repeatmasker.org/RepeatModeler.html, last accessed August 5, 2016), using default parameters. Deletions showing homology with a TE included in the database in at least 80% of their length or at both extremities (400 bp at the two ends) were classified accordingly.

Insertion dates of a set of complete *P. trichocarpa* LTR retrotransposons retrieved from http://www.agr.unipi.it/ricerca/plant-genetics-and-genomics-lab/sequence-repository (last accessed August 5, 2016) were estimated based on the amount of divergence between the 5′ and 3′ LTRs as previously described (SanMiguel et al. 1998). LTR sequences of each retrotransposon were recovered from the reference sequence and aligned with the *stretcher* command of the EMBOSS suite (Rice et al. 2000; Olson 2002). The evolutionary distance (*K*) between the two LTRs was calculated for each pairwise comparison with the *distmat* tool of EMBOSS, with the *nucmethod* option set to 2, in order to compute the distance measure with the Kimura’s Two-Parameter method (Kimura 1980). Finally, the time of insertion (T) was estimated for each retrotransposon with the substitution rate (*k*) of 4.72 2 × 10 ^−^ ^9^ (Cossu et al. 2011), via the following equation: *T* = *K*/2*k. Dated LTR retrotransposons were divided in two subsets: the first containing elements corresponding to detected deletions (SV-LTR) and the second containing those unrelated to deletions (noSV-LTR) and a difference between the two distributions of insertion dates was tested using the non-parametric two-sample Kolmogorov–Smirnov test.

### Detection of Transposable Elements Insertions

Insertions of transposable elements were detected exploiting the information carried by read pairs spanning the insertion site, i.e. pairs in which one read (referred to as “anchor” read) originates from the flanking regions of the inserted element and its mate (referred to as “mobile” read) originates from either the 5′ or the 3′ of the inserted element. “Anchor” reads are expected to be aligned to the reference genome and to create two clusters aligned in opposite orientation pointing toward the insertion site, while “mobile” reads are expected to be either not aligned or aligned in multiple positions of the genome (supplementary fig. S2, Supplementary Material online). The alignment of “mobile” reads against a database of transposable elements was used to classify the mobile elements causing each insertion. The database contained a set of known plant TEs, a set of poplar-specific repeat sequences and the sequences of the deletions detected in the present study that are likely related to TE activity (see Section 4 of supplementary materials, Supplementary Material online, for details). The analytical pipeline used to detect new transposable elements insertion sites is described in supplementary material (Supplementary Material online). The above-mentioned pipeline was used to detect new insertions with respect to the *P. trichocarpa* reference genome in the high-depth individuals and in the low-depth pool. Results obtained from the different samples were merged using a confidence interval of 250 bp around the breakpoints. The integrated list of insertions was analyzed with a custom python script to calculate the proportion of reads supporting the insertions in each sample and thus assign the genotype (non-carrier, heterozygous carrier, and homozygous carrier), using the same approach described for deletions (see Section 3 of supplementary material, Supplementary Material online, for details). TE insertions were considered to affect a gene when the entire interval in which the insertion was predicted overlapped an annotated gene.

### Detection of Genic CNVs

The detection of genic regions showing copy number variation was performed by exploiting the depth of coverage signature (DOC), including in the analysis only reads that were uniquely aligned to the reference genome. The tool *htseq-count* (Anders et al. 2014) was used to count the number of fragments aligned on each of the 41,335 *P. trichocarpa* annotated genes in the high-depth samples. Read counts were used as input data for the R package DESeq (v1.8.3), which is widely used for the detection of differential gene expression (Anders and Huber 2010). DESeq was employed to detect differentially covered genes between all the possible combinations of the seven high-depth samples. Data was normalized using the functions *estimateSizeFactors* and *sizeFactors* included in the DESeq package (Anders and Huber 2010). After dispersion estimation for each gene, a binomial test was used to identify differentially covered genes, following correction for multiple testing with the Benjamini–Hochberg procedure (Anders and Huber 2010). A corrected *P*-value < 0.05 was considered significant.

### Gene Ontology Annotation and Enrichment Analysis

The *P. trichocarpa* v3.0 gene annotation (Tuskan et al. 2006) was used to study genes affected by insertions, deletions, or CNVs. To obtain the Gene Ontology annotation, gene sequences were used as query for a BLASTx analysis against the Viridiplantae (taxid: 33090) non-redundant protein (nr) database. BLASTx results were imported into BLAST2GO version V.2.5.0 (Conesa et al. 2005) for the Gene Ontology (GO) assignment. The Annex tool implemented in BLAST2GO was used to improve the annotation by deriving terms due to verified links from “Molecular Function” terms to “Biological Process”, and “Cellular Component” ones. Annotation results were summarized through the mapping to the Plant GO-Slim, a reduced version of the Gene Ontology containing a selected number of nodes relevant for plants. Over- representation, compared to the rest of the genome, of GO terms in the subsets of genes included in the deletions, interrupted by insertions or showing differential coverage was tested using a Fisher’s Exact Test implemented in the Gossip package (Blüthgen et al. 2005) integrated in BLAST2GO. To reduce the number of false positives, a false discovery rate correction for multiple testing (Benjamini and Hochberg 1995) was applied and only differences with a corrected *P*-value <0.05 were selected.

### Sequence Evolution Analysis

The number of non-synonymous substitutions per non-synonymous site (d*N*), the number of synonymous substitutions per synonymous site (d*S*), and their ratios were calculated for a list of 30,055 genes orthologous between *P. trichocarpa* and *P. euphratica*. Orthologous genes were selected by a BLASTn (Altschul et al. 1990) analysis of the *P. trichocarpa* CDSs against the *P. euphratica* reference genome (GeneBank assembly accession: GCA_000495115.1). Only the portions of *P. trichocarpa* CDSs aligning for at least 150 consecutive bases to the *P. euphratica* genome were selected. Polymorphic positions between the two species were detected with a custom R function and the d*N*/d*S* ratio was calculated with the *kaks* function included in the R library “seqinr”. This function makes an unbiased estimate of the ratio of non-synonymous to synonymous nucleotide substitution for a set of aligned sequences (Li 1993). To assess the statistical significance of the difference in the d*N*/d*S* ratios observed between genes affected by structural variation and those not affected a randomization test was used. In brief, we contrasted the d*N*/d*S* ratios in genes affected by deletions, insertions, or genic CNVs to the distribution of means of the same feature in *s* = 10,000 randomly generated subsets of an equal number of genes drawn from the complete gene set. Let *q* be the number of times the mean value of the set of genes affected by structural variation was lower than the mean value of the randomly generated subset and *r* = *s*−*q*, the *P*-value has been calculated as the following: (*r* + 1)/(*s* + 1).

### Gene Expression Analysis

*P. trichocarpa* RNAseq data from four different tissues (developing xylem, leaf, callus from root, and cambium) was used to distinguish between experimentally verified and not experimentally verified genes. To this aim, expression levels were calculated from RNAseq data of each tissue as the absolute fragment values corrected by sequence length (FPKM values: the number of reads pairs per kilobase of transcript per million mapped reads) and genes were considered to be verified if in at least one of the four tissues the FPKM value was higher than zero. To assess the statistical significance of the difference in the expression levels observed between genes affected by structural variation and those not affected a randomization test was used. We contrasted the expression levels (calculated as the mean FPKM obtained in the four tissues) in genes affected by structural variation to the distribution of means of the expression levels calculated in *s* = 10,000 randomly generated subsets of an equal number of genes drawn from the complete gene set. Let *q* be the number of times the mean value of the set of genes affected by structural variation exceeded the mean value of the randomly generated subset and *r* = *s*−*q*, the *P*-value has been calculated as the following: (*r* + 1)/(*s* + 1).

### Characterization of SV

Different statistical tests have been applied to study how INDELs and genic CNVs are distributed within the poplar genome.

- To test for preferential localization of INDELs outside of (i) genes, (ii) individual genic portions (CDSs, 5′-UTRs, 3′-UTRs, and introns), or (iii) at a distance greater than 500 bp from genes, a randomization test was used. One hundred simulated datasets of deletions and insertions were obtained by transferring the detected variants to new randomly assigned genomic location and the number of affected genes was computed. Confidence intervals of the number of affected genes were based on empirical distribution.
- Preferential localization of INDELs in the centromeric regions of poplar chromosomes was tested against the null hypothesis that INDELs follow a Poisson distribution with mean equal to the mean number of INDELs per 250 kb windows. The resulting *P*-values were corrected for multiple testing (Benjamini and Hochberg 1995).
- The relationship between the number of INDELs and repetitiveness was assessed by comparing the distribution of 20-mers in 250 kb windows not carrying INDELs with that in windows carrying one or more INDELs for insertions and deletions separately. Significance was tested using Wilcoxon–Mann–Whitney test and *P*-value was corrected for multiple testing (Benjamini and Hochberg 1995).
- To test whether the distance between genic CNVs and INDELs was shorter than that between all genes and INDELs, Wilcoxon–Mann–Whitney test was used.
- Significance of the clustering of genic CNVs was assessed by comparing the number of clustered genic CNVs (clusters were defined as groups of 2 or more genic CNVs in a single window) observed in our data with the number of clustered genic CNVs obtained by simulating 10,000 times a random distribution of genic CNVs across the genome.
- Wilcoxon–Mann–Whitney test was used to test the hypothesis that windows with genic CNVs clusters have a higher number of genes compared with windows showing no genic CNVs.
- To test for a preferential localization of genic CNVs along the poplar chromosomes we used a chi-square test: expectation was based on the null hypothesis that genic CNVs have the same distribution of genes and was computed as the total number of genic CNVs multiplied by the proportion of genes falling in the same distance bin. Distance was measured in bins of 1 Mb. Distances greater than 10 Mb were pooled.
- Local enrichment of genic CNVs in inter- or intra-specific comparisons was computed in 2 Mb windows assuming as null hypothesis a Poisson distribution in which the number of genic CNVs per window is equal to the average number of genic CNVs per window in the chromosome (to allow for heterogeneity between chromosomes).
- Local enrichment of genic CNVs belonging to each GO category over-represented in genic CNVs was tested in 250 kb windows assuming a Poisson distribution in which the null expectation is the genome-wide average number of genic CNVs per window for each category. The obtained *P*-value was corrected for multiple testing (Benjamini and Hochberg 1995). The same approach was used to test for enrichment of all genes belonging to the over-represented GO categories.

## Supplementary Material

Supplementary figures S1–S17 and tables S1–S13 are available at *Molecular Biology and Evolution* online (http://www.mbe.oxfordjournals.org/).

Supplementary Data
