# Supplementary material for: Characterization of the Poplar Pan-Genome by Genome-Wide Identification of Structural Variation
Source: Mol Biol Evol. 2016 Aug 7;33(10):2706–19. doi: 10.1093/molbev/msw161 (PMC5026262; doi:10.1093/molbev/msw161)
Supplement: Supplementary Data [file supp_33_10_2706__index.html]

Characterization of the poplar pan-genome by genome-wide identification of structural variation. — Characterization of the Poplar Pan-Genome by Genome-Wide Identification of Structural Variation — Characterization of the Poplar Pan-Genome by Genome-Wide Identification of Structural Variation — Supplementary Data 

# Characterization of the Poplar Pan-Genome by Genome-Wide Identification of Structural Variation

## Supplementary Data

files

- Supplementary Data - docx file
- Supplementary Data - xlsx file
