# Supplementary material for: Characterization of the Poplar Pan-Genome by Genome-Wide Identification of Structural Variation
Source: Mol Biol Evol. 2016 Aug 7;33(10):2706–19. doi: 10.1093/molbev/msw161 (PMC5026262; doi:10.1093/molbev/msw161)
Supplement: Supplementary Data [file supp_msw161_Supplementary_material_v12.docx]

# SUPPLEMENTARY INFORMATION

## 1) Simulated datasets

To investigate the sensitivity in the detection of INDELs and to choose the best performing tools and parameters, one thousand insertions and deletions were simulated by modifying the *P. trichocarpa* v3 reference genome. Variants were simulated by randomly removing one thousand of repeated sequences (ranging from 1 to 25 kb in length) from their original position in the *P. trichocarpa* v3 reference genome and inserting them in a new randomly chosen position of the genome. Thus, by aligning the original *Nisqually-1* reads to the modified genome, simulated insertions were expected where the 1000 sequences have been removed from the original reference, while deletions were expected where the sequences have been inserted (Supplementary Figure 18).

To perform the detection of the simulated INDELs, three different datasets of short reads have been employed. The first dataset included the original short reads obtained from the *P. trichocarpa* reference genotype *Nisqually-1*. This dataset allowed studying the ability in detecting INDELs in the presence of possible library and sequencing bias that could occur in a real dataset. However, when analyzing the real reads of *Nisqually-1*, a genotype characterized by high levels of heterozygosity and haplotype diversity (Kelleher et al. 2007), a number of heterozygous variants not included in the reference genome (which is a combination of the two possible haplotypes) is expected to be found. Thus, by using the real reads obtained from *Nisqually-1*, an accurate estimation of the false positives was not possible. For this reason a second dataset consisting of reads simulated from the original *P. trichocarpa* v3 reference genome was also employed. Approximately 70 million of paired reads 100 bp long were simulated using *wgsim* (https://github.com/lh3/wgsim), by setting the indel rate to 0%, the error rate to 0.01 and the outer distance between the two read ends to 420 bp. This dataset was employed to obtain a more realistic estimation of the false positives in the detection of INDELs. Finally, a third dataset was simulated with the aim to study our ability in assigning the correct genotype to the detected INDELs. Thus, we created a simulated dataset by joining 35 million paired reads simulated from the original *P. trichocarpa* v3 reference genome with other 35 million simulated from the modified reference genome. Reads were simulated using *wgsim* (https://github.com/lh3/wgsim) as previously described. Since half of the reads came from the modified reference and the other half came from the original one, in this dataset the simulated structural variants resulted heterozygous.

The reads of the three datasets were aligned to the modified reference using BWA (Li and Durbin 2009) with default parameters. Library and coverage statistics of the three datasets are reported in Supplementary Table 14.

## 2) Identification of simulated deletions

### *Parameters of compared tools*

We used the simulated set of variants to study the performance of four different available tools in detecting deletions: CLEVER (Marschall et al. 2012), DELLY (Rausch et al. 2012), GASV (Sindi et al. 2009) and Pindel (Ye et al. 2009). The commands we used for each tool are as following (we have specified the mean and standard deviation insert size of each dataset if required):

CLEVER (version 2.0rc3): we used the option “use_xa” that allows the interpretation of XA tags in the input BAM file reported by the aligner BWA. From the vcf output file we selected only the deletions ranging from 1 to 25 kb in size supported by at least 5 paired-ends.

DELLY (version 0.3.3): the parameters were default values. The vcf output was filtered in order to select only called deletions from 1 to 25 kb in size and supported by at least 5 paired-ends having a median mapping of at least 20.

GASV (version 2.0): we set the option “minClusterSize” to 5 which allows GASV to detect variants supported by at least five reads. GASV output file was filtered to select only deletions ranging from 1 to 25 kb in size and to remove variants in which the tool wasn’t able to detect a single deletion explaining the data (flagged by a value of -1 in the field “Localization” of the output table that reports the square root of the breakpoint region).

Pindel (version 0.2.5a3): we set "Maximum event size index" to 5 which allows Pindel to detect events whose size are up to 32,368 and “Minimum support for event” to 5 which allows the detection of variants supported by at least five reads. From the output file we selected only deletions ranging from 1 to 25 kb in size and supported by reads having a mean mapping quality of at least 20.

### *Simulation results*

Deletions detected by the different tools were classified as true positives (TPs) if they overlapped the simulated ones with a difference in the estimated breakpoints smaller than 250 bp. Otherwise deletions were classified as false positives (FPs). False negatives (FNs) were calculated as simulated deletions not detected by the employed tools. Precision was calculated as the fraction of TPs over all predictions. Results obtained with the four tools on the real and simulated *Nisqually-1* datasets are reported respectively in Supplementary Table 15 and Supplementary Table 16. On real dataset CLEVER predicted a significant smaller number of deletions with respect to the other three tools. GASV was the tool that predicted the higher number of TPs and performed with the higher precision; DELLY performed only slightly worse than GASV while Pindel detected a considerable smaller number of TPs and performed with the lower precision. On the contrary, Pindel obtained the best accuracy in the prediction of the breakpoints of the deletions, followed soon after by DELLY, while CLEVER and GASV resulted to be less accurate. The high number of FPs detected by all the tested tools and, as a consequences, the low precision is likely a consequence of the high levels of heterozygosity and haplotype diversity in *Nisqually-1*. With high probability, most of the deletions detected as FPs are true heterozygous variants of *Nisqually-1* that haven’t been included in the haplotype represented in the reference genome. This hypothesis was confirmed by the estimation of the frequencies of the detected deletions (see section “Genotype calling”): as expected, TPs resulted to be homozygous (with a mean frequency very close to one) while FPs resulted to be heterozygous (mean frequency ~0.5).

To obtain a more reliable estimation of FPs we thus analyzed a simulated dataset in which only the simulated deletions were supposed to be detected (Supplementary Table 16). In the simulated dataset, the tool showing the best performance in terms of number of detected true positives and precision was DELLY. CLEVER and GASV showed a comparable precision but detected a significant lower number of TPs while Pindel performed worse for both parameters. In terms of accuracy in estimating the breakpoints of the deletions, results were consistent with those obtained by analyzing the real dataset: Pindel showed the most accurate estimation, followed soon after by DELLY, while CLEVER and GASV obtained the worst performance.

Considering the results obtained in both datasets, we choose DELLY as the best performing tool for the detection of deletions in our data. We studied if the integration of DELLY predictions with those obtained by the other three tools could improve the results. Prediction obtained with DELLY were integrated with those obtained by each of the other three tools by combining overlapping deletions having a difference in the estimated breakpoints smaller than 250 bp. Considering the high accuracy of DELLY in estimating the breakpoints of the deletions, to the integrated deletions we assigned the breakpoints called by DELLY. In both datasets the pair DELLY-GASV was the one showing the highest accuracy.

### *Genotype calling*

To estimate the genotype of the detected deletions a custom python script employed. For each deletion, the script counts from the alignment BAM file the number of reads supporting the variant allele (i.e. read pairs aligned with an anomalously high distance spanning the predicted deletion) and the number of those supporting the reference genotype (i.e. reads pairs aligned at the expected distance and spanning one of the two breakpoints of the deletions). The ratio between the reads supporting the deletion and the total number of reads spanning the breakpoints (i.e. reads supporting the deletion and those supporting the reference allele) was used to estimate the frequency of the variant in the dataset and to assign the genotype. For each variant, the genotype was predicted only in samples where the breakpoints of the variant were covered by at least five reads.

To study the accuracy in estimating the genotype of the deletions, we run the script on the simulated dataset obtained by mixing a set of reads simulated from the original *P. trichocarpa* reference genome with a set obtained from the modified reference genome. Using this dataset the one thousand of deletions were expected to be heterozygous. The tools DELLY and GASV were run on the obtained BAM file as described previously and results of the two tools were integrated. 940 out of the one thousand simulated deletions were detected with a precision of 100 %. In detected deletions we obtained a mean frequency value of 0.52 with a standard deviation of 0.08.

## 3) Identification of simulated insertions

We used the simulated datasets to evaluate our ability in the detection of insertions caused by the activity of transposable elements. To this aim, the alignment of the original and the simulated *Nisqually-1* reads against the modified reference was analyzed with a custom pipeline (see section 4 for a detailed description of the pipeline). Insertions detected by the pipeline were classified as true positives (TPs) if the insertion sites were predicted less than 250 bp apart from the simulated ones. Otherwise insertions were classified as false positives (FPs). False negatives (FNs) were calculated as simulated insertions missed by the pipeline. Precision was calculated as the fraction of TPs over all predictions. Using the original *Nisqually-1* reads we detected 766 out of the 1000 simulated insertions, with a precision of 62.3% while in the simulated dataset we detected 877 TPs with a precision of 100% (Supplementary Table 17).

As for deletions, we used a custom python script to estimate the genotype of the detected insertions by dividing the number of reads supporting the insertion by the total number of paired reads spanning the insertion site. Reads supporting the insertion were defined as read pairs in which one read was aligned within the 500 bp flanking the insertion site and its mate was either not aligned or aligned in another genomic and showed homology with the 5’ or 3’ of the inserted sequence. To study the accuracy of the script in estimating the genotype of the insertions, the script was run on the dataset containing heterozygous simulated variants.

837 out of the one thousand of heterozygous simulated insertions were detected with a precision of 100%, a mean frequency of 0.59 and a frequency standard deviation of 0.12.

## 4) Identification of transposable element insertions

The detection of transposable elements insertions was performed by exploiting the information carried by read pairs spanning the insertion site, i.e. pairs in which one read (referred to as “anchor” read) originates from the flanking regions of the inserted element and its mate (referred to as “mobile” read) originates from either the 5’ or the 3’ of the inserted element. “Anchor” reads are expected to be aligned to the reference genome and to create two clusters aligned in opposite orientation pointing toward the insertion site, while “mobile” reads are expected to be either not aligned or aligned in multiple positions of the genome. The pipeline used to detect new transposable elements insertion sites is composed by four main steps (Supplementary Figure 2):

1. The forward and reverse oriented “anchor” reads (red and blue arrows) were extracted from the alignment files and separately *de novo* assembled using CAP3 (Huang and Madan 1999) in order to obtain two consensus sequences for each putative insertion site flanking regions (red and blue bars). CAP3 was run by setting an overlap length cutoff of 16 (option o), a clipping range of 6 (option y), an overlap similarity score cutoff of 251 (option s), a maximum overhang percent length of 100 (option h), a match score factor of 40 (option m), a segment pair score cutoff of 21 (option i) and a chain score cutoff of 31 (option j).

2. Consensus sequences of the insertion sites flanking regions were aligned to the *Populus trichocarpa* reference genome using BLASTn (Altschul et al. 1990). Putative insertion sites (black lightning) were called in regions flanked by two consensus sequences aligned at a distance lower than the mean insert size of the sequenced library and derived from “anchor” reads having an opposite orientation and pointing toward the insertion site.

3. To reconstruct the two ends of the inserted element, the mates of the “anchor” reads (green arrows) used to predict a possible insertion site were selected and *de novo* assembled using CAP3.

4. To confirm the TE insertion, consensus sequences obtained by the assembly of the “mobile reads” (green bars) were aligned against a database of transposable elements using BLASTn. The database included the plant section of the RepBase18.09 database (Jurka et al. 2005), a list of *Populus trichocarpa* LTR retrotransposons retrieved from <http://www.agr.unipi.it/ricerca/plant-genetics-and-genomics-lab/sequence-repository.html>, a set of poplar repeat sequences *de novo* detected and classified by analyzing the *Populus trichocarpa* reference genome with *RepeatModeler* (<http://www.repeatmasker.org/RepeatModeler.html>) and the sequences of the deletions detected in the present study. New transposable element insertions were called when the reconstructed ends of the putative inserted sequenced were aligned at the two ends of a same TE included in the database.

## 5) Experimental validation of INDELs

A PCR-based assay was designed to experimentally validate a set of identified insertions and deletions. For each variant four primers were designed and were combined in 3 primer pairs (Supplementary Figure 19): one pair (1-2) amplifying the 5' junction between the deleted/inserted sequence and the reference sequence, one pair (3-4) amplifying 3' junction, and a third pair (1-4) connecting the two genomic regions flanking the variant. Deletions are confirmed by the amplification of the two external primers (1-4), while insertions are confirmed by the amplification of the two junctions (1-2 and 3-4). Primer design was performed using BatchPrimer3 (You et al. 2008). DNA amplifications were performed in 15 µl PCR reactions, using KAPA2G Fast Hot Start Ready Mix (Kapa Biosystems). The reactions were performed in the Geneamp 9700 PCR system (Applied Biosystems, Foster City, CA), under the following conditions: 95 °C for 2 minutes, 35 cycles of 15 seconds at 95 °C, 15 seconds at 56 °C and 15 seconds at 72 °C, followed by a final extension of 1 minute at 72 °C. Amplification results were run on a 1% agarose gel.

The assay was used to experimentally validate a set of 29 randomly selected variants. PCR assays confirmed 14 out of the 16 tested insertions (Supplementary Figure 3) and all the 13 tested deletions (Supplementary Figure 4), with a sensitivity of 88% and 100% respectively. Only for one tested variant the genotype obtained by PCR assays was in disagreement with that called by the detection tools: an insertion called in heterozygous state resulted homozygous in the PCR assay. The list of the tested variants together with primers sequences and PCR conditions are reported in Supplementary Table 18.

## 6) Integration of INDELs with de novo assembly

A *P. nigra de novo* assembly obtained from Illumina short reads of the sample *Poli* was used to perform an *in silico* validation of the insertions and deletions detected in that sample.

*P. nigra* *de novo* assembly was produced using the software CLC Genomics Workbench v4.7 (http://www.clcbio.com). Prior to assembly, *Poli* paired-end reads were filtered for chloroplast and mitochondrial contaminations using rNA (Vezzi et al. 2012) with default parameters and trimmed for quality using CLC with default parameters (http://clcsupport.com/clcgenomicsworkbench/600/index.php?manual=Automatic_trimming.html). 378.7 M reads were assembled in 104,431 contigs with N50 and NG50 of 6,130 and 4,434 bp respectively. The total assembly length corresponded to 339.5 Mb with a mean contig length of 3,251 bp.

To validate deletions, 500 bp upstream and downstream each called variant were extracted from the *P. trichocarpa* reference genome and aligned to the *de novo* assembly of *Poli* using BLASTn (Altschul et al. 1990) with an *E*-value threshold of 10^-20^. Only variants for which both up- and downstream regions were aligned in the same contig of the assembly and with the expected orientation (i.e. variants entirely included in a single contigs of the *de novo* assembly) were retained for the validation analysis. A deletion was validated if the alignment distance of the two surrounding regions on *P. nigra* resulted at least 500 bp smaller than the distance on the *P. trichocarpa* reference genome. On the other hand, to validate insertions, an alignment distance of at least 500 bp greater than the original distance was required. Out of the 3,497 deletions detected in *Poli* 2,393 (68.4%) were located in the assembly satisfying our criteria for inclusion in the analysis. In 2,355 of them (98.4%), the assembly confirmed the presence of a deletion of at least 500 bp, with a correlation between the deletion length predicted by the detection tools and that calculated on the assembly of 0.99. Results were different when analyzing the insertions: only 421 (8.4%) out of the 4,985 insertions detected in *Poli* were located in the assembly and only 76 (18%) were confirmed as insertions of at least 500 bp with respect to the *P. trichocarpa* reference genome. Other 64 variants were confirmed as insertions of at least 100 bp. For both deletions and insertions, the mean frequency of the variants confirmed by the *P. nigra* assembly (0.97 for both deletions and insertions) was significantly higher than the mean frequency of the unconfirmed variants (0.62 and 0.73 respectively for deletions and insertions) (Supplementary Figure 20). Thus, the majority of INDELs not confirmed by the *de novo* assembly are probably heterozygous variants for which only the allele without the variant has been included in the *de novo* assembly. These results suggested that the use of a *de novo* assembly to detect or validate INDELs could be of valuable use in the case of deletions but less effective for insertions. In fact, one of the main problems of the *de novo* assembly process using short reads is the management of repeated sequences, such as those resulting from transposable elements insertions. As a consequence, in proximity to TE insertions the assembly has a high probability to be fragmented (i.e. the inserted sequence is not entirely reconstructed) or, in the case of heterozygous insertions, to include the allele without the insertion.

## 7) Performance of INDELs detection

For the detection of INDELs we exploited the paired-end mapping information generated from next-generation sequencing data. Deletions were detected using a combination of the two available tools DELLY (Rausch et al. 2012) and GASV (Sindi et al. 2009) while insertions were detected using a specifically developed pipeline. Simulations showed a high reliability of the two employed approaches: using real reads we obtained a sensitivity of 0.84 and 0.77 in detecting deletions and insertions respectively, while using simulated reads the sensitivity increased to 0.96 and 0.88 respectively. Considering that both approaches are based on the alignment properties of the paired reads flanking the variants, one possible factor affecting the performance of both methods is the presence in the flanking regions of INDELs of sequences that are difficult to map, such as repetitive sequences.

Using the simulated reads we obtained a false discovery rate (FDR) of 0.10% and 0% for deletions and insertions respectively while using real reads the FDR couldn’t be calculated due to the impossibility to distinguish between false positives and real heterozygous variants originally presents in the sample from which reads have been generated. However, an estimation of FDR using real reads has been obtained with the experimentally PCR-based validation of a random set of identified variants: a FDR of 6.25% has been obtained for the two methods combined. The most significant source of false positives arises from alignment artifacts in short-read data. Thus, a large fraction of false positives is probably located in regions that are difficult to map, such as repetitive regions. In this study, we used relatively small insert sizes (200-500 bp); using larger insert sizes has the advantage of greater genomic coverage per sequenced fragment but increases the difficulty of breakpoint annotation. It is likely that complete characterization of all variants will require paired-end sequencing of several libraries of different insert sizes, allowing reads to fall outside any repetitive sequence present near the breakpoints. The reliability of the detected INDELs was also confirmed by the mapping of the variants detected in the sample *Poli* to a whole genome *de novo* assembly obtained using the reads of the same sample. 68.4% of deletion sites and only 8.4% of insertion sites were localized in the *P. nigra* assembly by the alignment of the INDELs surrounding regions. This difference is probably due to the fact that repetitive sequences, such as transposable elements, are difficult to assemble using NGS short reads; thus in proximity of TE insertions it’s more probable that the contiguity of the assembly is interrupted while this problem is not present in proximity of deletions. This result is a first indicator of the reliability of called insertions. 98.4% of mapped deletions were confirmed by the assembly while the percentage of confirmed insertions was considerably lower (18%). This could be explained by two main reasons: 1) due to their repetitive nature, TE sequences are difficult to assemble thus assembly error could occur in correspondence to insertions; 2) in correspondence to heterozygous insertions, the haplotype without the insertion, which is the easier to assemble, is preferred. The second hypothesis is confirmed by the higher frequency of the variants confirmed by the *P. nigra* assembly with respect to the unconfirmed ones (Supplementary Figure 20).

## 8) Localization of poplar centromeres

To estimate the position of poplar centromeres we exploited the distribution of the gene density along the nineteen chromosomes. In fact genes are thought to be relatively rare in the centromeres of higher eukaryotes (Copenhaver 1999). To this aim, we divided each chromosome in non-overlapping windows of 250 kb, we calculated the number of genes annotated in each window and we selected as the putative centromeric region the window having the lower gene density.

# LITERATURE CITED

Altschul SF, Gish W, Miller W, Myers EW, Lipman DJ. 1990. Basic local alignment search tool. J. Mol. Biol. 215:403–410.

Copenhaver GP. 1999. Genetic Definition and Sequence Analysis of Arabidopsis Centromeres. Science (80-. ). 286:2468–2474.

Huang X, Madan A. 1999. CAP3: A DNA sequence assembly program. Genome Res. 9:868–877.

Jurka J, Kapitonov V V, Pavlicek A, Klonowski P, Kohany O, Walichiewicz J. 2005. Repbase Update, a database of eukaryotic repetitive elements. Cytogenet. Genome Res. 110:462–467.

Kelleher CT, Chiu R, Shin H, Bosdet IE, Krzywinski MI, Fjell CD, Wilkin J, Yin T, DiFazio SP, Ali J, et al. 2007. A physical map of the highly heterozygous Populus genome: integration with the genome sequence and genetic map and analysis of haplotype variation. Plant J. 50:1063–1078.

Li H, Durbin R. 2009. Fast and accurate short read alignment with Burrows-Wheeler transform. Bioinformatics 25:1754–1760.

Marschall T, Costa IG, Canzar S, Bauer M, Klau GW, Schliep A, Schönhuth A. 2012. CLEVER: clique-enumerating variant finder. Bioinformatics 28:2875–2882.

Rausch T, Zichner T, Schlattl A, Stütz AM, Benes V, Korbel JO. 2012. DELLY: structural variant discovery by integrated paired-end and split-read analysis. Bioinformatics 28:i333–i339.

Sindi S, Helman E, Bashir A, Raphael BJ. 2009. A geometric approach for classification and comparison of structural variants. Bioinformatics 25:i222–30.

Vezzi F, Del Fabbro C, Tomescu AI, Policriti A. 2012. rNA: a fast and accurate short reads numerical aligner. Bioinformatics 28:123–124.

Ye K, Schulz MH, Long Q, Apweiler R, Ning Z. 2009. Pindel: a pattern growth approach to detect break points of large deletions and medium sized insertions from paired-end short reads. Bioinformatics 25:2865–2871.

You FM, Huo N, Gu YQ, Luo M-C, Ma Y, Hane D, Lazo GR, Dvorak J, Anderson OD. 2008. BatchPrimer3: a high throughput web application for PCR and sequencing primer design. BMC Bioinformatics 9:253.

# SUPPLEMENTARY FIGURES


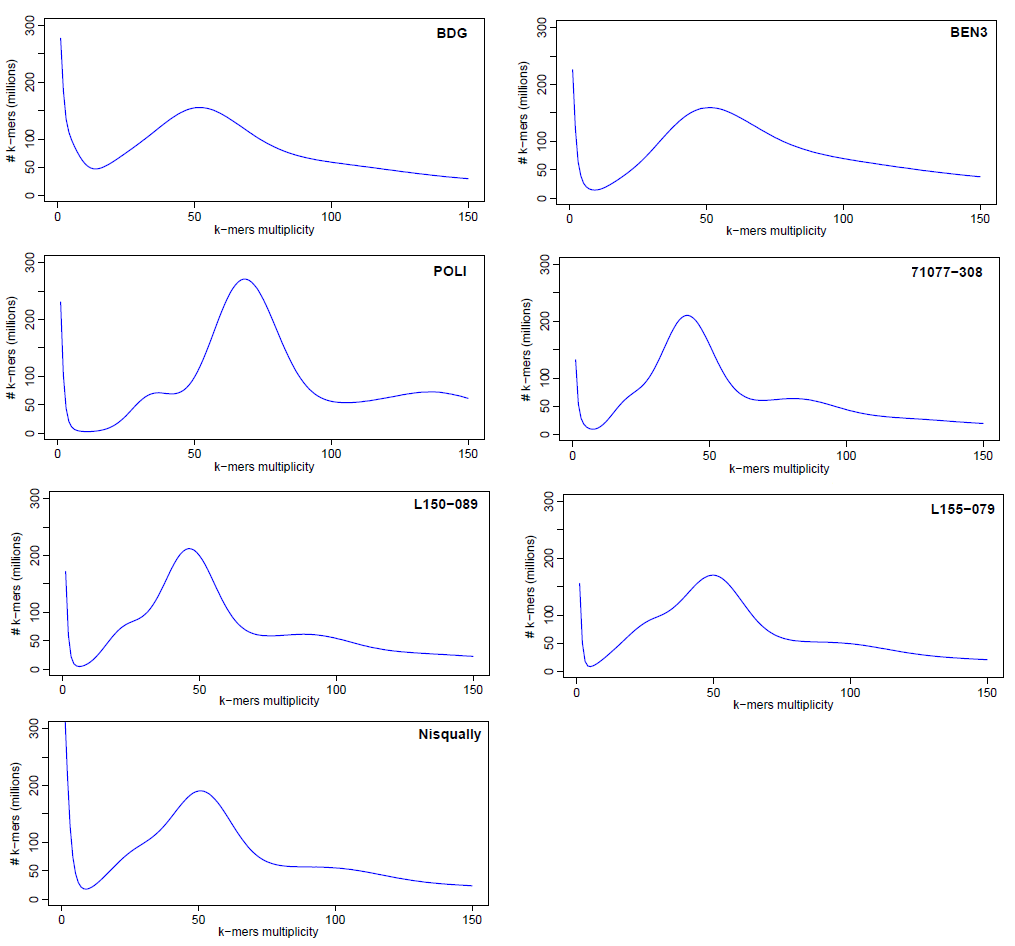


**Figure S-1**. 16-mer multiplicity calculated on the short reads of each resequenced sample.


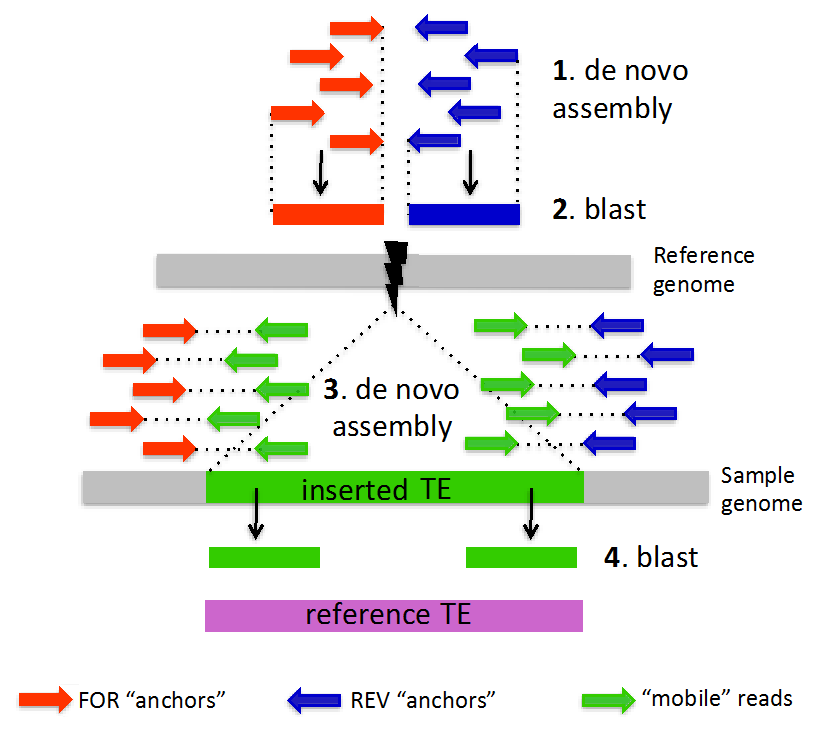


**Figure S-2.** Graphical representation of the pipeline developed for the detection of insertions.


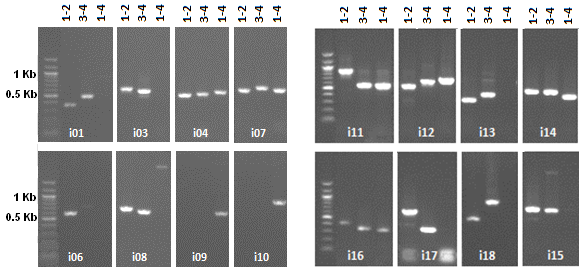


**Figure S-3.** Patterns of PCR amplifications obtained for the tested insertions. Amplification of primer pairs 1-2 and 3-4 confirms the insertions while the pair 1-4 amplifies when there is no insertion. Amplification of all three primer pairs is expected when the insertion is heterozygous.


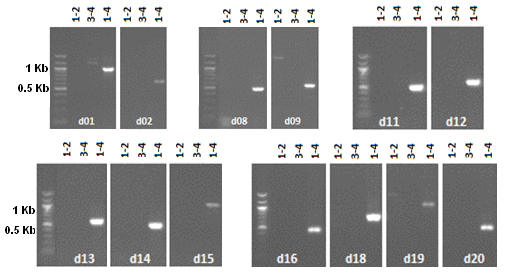


**Figure S-4.** Patterns of PCR amplifications obtained for the tested deletions. Primer pairs 1-2 and 3-4 amplify when the sequence is not deleted, while an amplification of the pair 1-4 confirms the deletion.


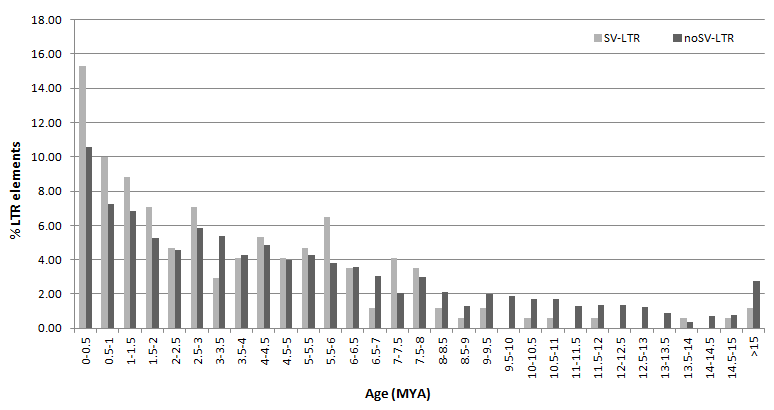


**Figure S-5.** Insertion age of LTR retrotransposons involved in structural variation (SV-LTR) and of those not involved in SV (noSV-LTRs). The non‑parametric two-sample Kolmogorov‑Smirnov test was used to test the null hypothesis that the two classes originated from the same distribution.

**Figure S-6.** Length distribution of detected deletions and insertions.

**Figure S-7.** INDELs frequency in the P. nigra low-depth pool.

**Figure S-8.** Repetitiveness (measured as the mean 20-mer number) in 250 kb windows as a function of the number of detected insertions (left) or deletions (right). Significance was tested using Wilcoxon test and p-values were corrected for multiple testing.

**Figure S-9.** Distribution of the distance between deletions (left) and insertions (right) and the nearest annotated gene (light blue) or genic CNV (blue). Mann-Whitney-Wilcoxon Test P-values < 2*10^-16.


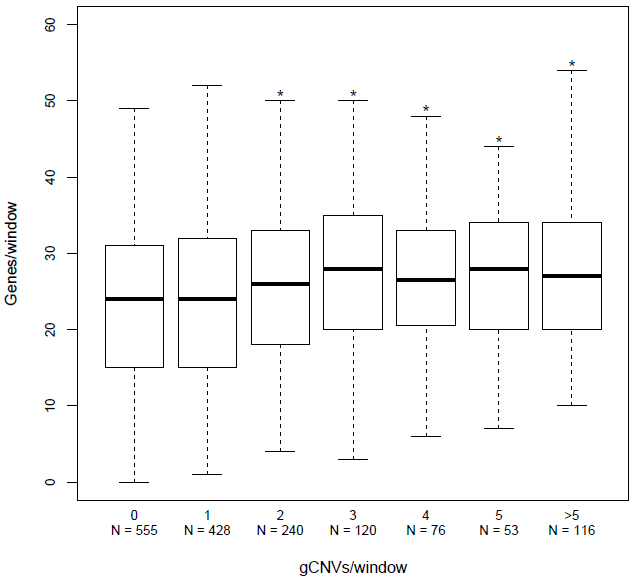


**Figure S-10.** Distributions of the number of annotated genes in 250 kb windows in which 0, 1, 2, 3, 4, 5, or more than 5 genic CNVs have been detected. N = number of 250 kb windows in which the corresponding number of genic CNVs has been detected. *: Number of genes per window significantly higher compared to windows with N=0 (Wilcoxon-Mann-Whitney p<0.05)

**Figure S-11.** Distance of genic CNVs from the nearest telomere (top) and centromere (bottom). *: p< 0.05, **: p< 0.01.


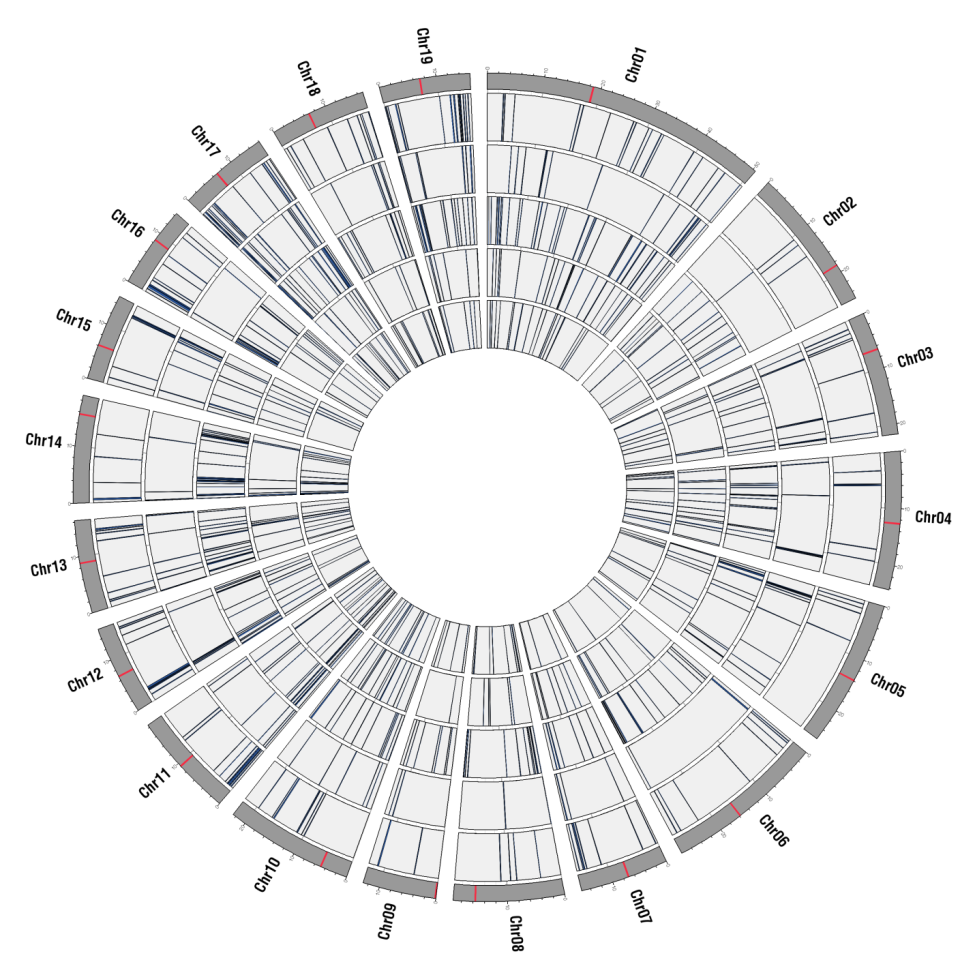


**Figure S-12**. **Genomic localization of inter- and intra-species specific genic CNVs**. From outer to inner layer: *P. nigra* intraspecific genic CNVs, *P. deltoides* intraspecific genic CNVs, *P. nigra-P. deltoides* specific genic CNVs, P*. nigra*-*P. trichocarpa* specific genic CNVs and *P. deltoides*-*P. trichocarpa* specific ones.


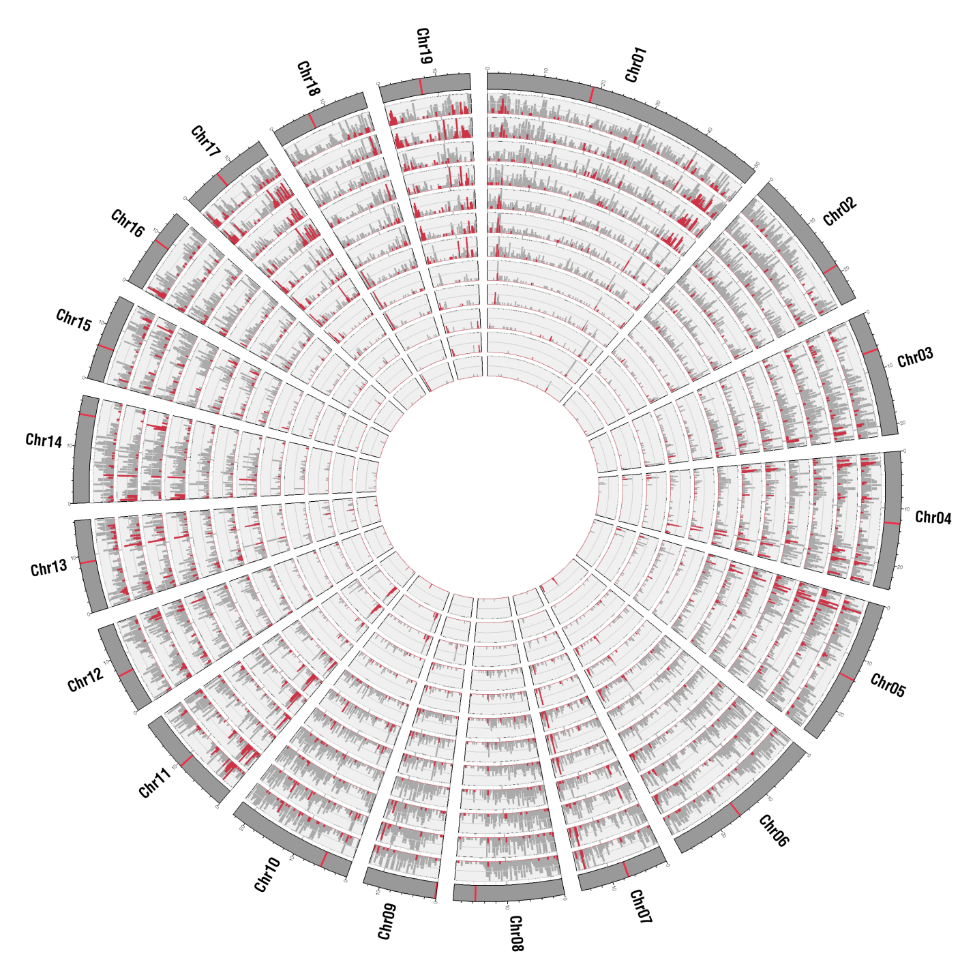


**Figure S-13.** Genomic distribution of all genes (grey) and genic CNVs (red) belonging to each of the twelve enriched GO categories. GO categories from outer to inner: 1) transferase activity, 2) nucleotide binding, 3) hydrolase activity, 4) response to stress, 5) cellular protein modification process, 6) kinase activity, 7) signal transduction, 8) receptor activity, 9) signal transducer activity, 10) carbohydrate binding, 11) pollen-pistil interaction, 12) cell-cell signalling.

**Figure S-14. Proportion of non-expressed genes calculated separately for homozygous (hom) and heterozygous (het) INDELs.** The proportion of non-expressed genes was significantly higher in genes affected by homozygous deletions and insertions than the whole transcriptome (chi-square p-values: 8.3E-08 and 1.34E-09 respectively).

**Figure S-15.** Expression levels in the whole transcriptome and in genes affected by SV calculated separately for each studied tissue.

**Figure S-16.** Rates of nonsynonymous to synonymous changes (dN/dS) in all genes affected by deletions (left) and insertions (right) and in genes in which the INDEL specifically disrupt CDSs, introns, 5’ UTRs and 3’ UTRs. Significance of the difference of dN/dS values between all affected genes and the four categories was tested using a randomization test.

**Figure S-17.** Variation in size of the Populus dispensable genome as a function of the number of analyzed samples.


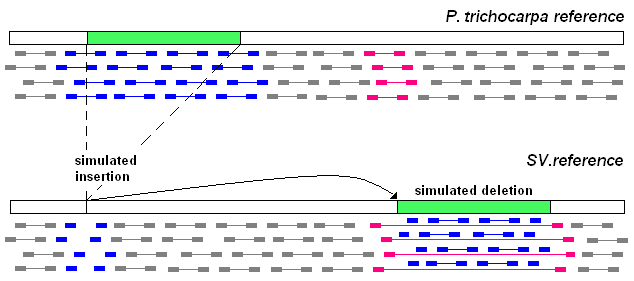


**Figure S-18.** Representation of the strategy employed to simulate one thousand insertions and

deletions in the *P. trichocarpa* v3 reference genome. One thousand sequences 1-25 kb long (green bars) were excised from the *P. trichocarpa* reference genome and randomly inserted in a new location of the genome. Simulated insertions were expected in the regions from which the sequences were removed while simulated deletions were expected in the regions were the sequences have been moved.


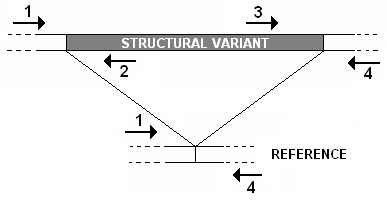


**Figure S-19**. Representation of the PCR assay performed to validate detected insertions and deletions. Homozygous deletions are confirmed by the amplification of the primer pair 1-4 and the non-amplifications of primer pairs 1-2 and 3-4 while homozygous insertions are confirmed by the amplification of primer pairs 1-2 and 3-4 and the non-amplification of the primer pair 1-4. Heterozygous variants are confirmed by the amplification of all the three primer pairs.

**Figure S-20.** Boxplots representing the distribution of the frequencies in deletions (blue) and insertions (light blue) validated (left) and not validated (right) by the de novo assembly.

# SUPPLEMENTARY TABLES

**Supplementary Table 1. DNA libraries and coverage statistics of high-depth samples.** List of all the *P. nigra* (N), *P. deltoides* (D) and *P. trichocarpa* (T) resequenced accessions with the corresponding library and coverage statistics obtained after the alignment to the *P. trichocarpa* reference genome.

| **Sample** | **Species^a^** | **Filtered reads^b^** | **Sequencing depth^c^** | **Read length^d^** | **Genome coverage^e^** | **Genome coverage >10X^f^** | **Gene coverage^g^** | **Insert size^h^** | **SRA^i^** |
| --- | --- | --- | --- | --- | --- | --- | --- | --- | --- |
| Nisqually-1 | T | 279.6 | 41.7 | 72.99 | 97% | 95% | 99.0% | 224 | SRR1762393, SRR1762405 |
| L150-089 | D | 206.8 | 30.9 | 98.96 | 83% | 62% | 96.3% | 373 | SRS1320691 |
| L155-079 | D | 196.3 | 30.0 | 94.64 | 82% | 60% | 96.2% | 425 | SRS1328499 |
| BDG | N | 288.5 | 28.2 | 97.38 | 81% | 57% | 96.1% | 447 | SRS1218639 |
| 71077-308 | N | 211.6 | 27.2 | 92.29 | 84% | 63% | 96.5% | 465 | SRS1218640 |
| Poli | N | 323.5 | 45.7 | 99.5 | 82% | 68% | 96.0% | 409 | SRS1218642 |
| BEN3 | N | 284.8 | 26.2 | 96.98 | 82% | 60% | 96.1% | 383 | SRS1218641 |

^a^Sample species: N= *Populus nigra*, D *=* *Populus deltoids,* T = *Populus trichocarpa*. ^b^Million of reads left after the quality trimming and contaminant filtering process. ^c^Mean sequencing depth calculated considering only the uniquely aligned reads. ^d^Mean read length obtained after quality trimming. ^e^Percentage of *P. trichocarpa* reference genome covered by at least one sequenced read. ^f^Percentage of *P. trichocarpa* reference genome covered by at least ten sequenced reads. ^g^Percentage of *P. trichocarpa* genic sequences covered by at least one sequenced read.  ^h^Mean library insert size. ^i^Short Read Archive accessions.

**Supplementary Table 2. Libraries and coverage statistics of *P. nigra* low-depth samples.**

| **Sample** | **M raw reads^a^** | **M filtered reads^b^** | **Sequencing depth^c^** | **Read length^d^** | **Genome coverage^e^** | **Insert size^f^** | **SRA** |
| --- | --- | --- | --- | --- | --- | --- | --- |
| 92525-25 | 76.0 | 26.0 | 5.3 | 99.76 | 61% | 296 | SRS1218634 |
| 1-A10 | 78.6 | 26.7 | 5.2 | 99.69 | 62% | 281 | SRS1218635 |
| 6-A06 | 85.1 | 29.0 | 5.2 | 98.14 | 63% | 308 | SRS1218637 |
| NVHOF2/19 | 61.4 | 23.5 | 5.3 | 97.13 | 52% | 272 | SRS1218628 |
| 6-A23 | 64.2 | 24.7 | 5.2 | 95.07 | 60% | 286 | SRS1218629 |
| SRZ | 70.6 | 28.3 | 5.2 | 96.55 | 62% | 285 | SRS1218631 |
| VGN | 63.6 | 26.1 | 5.2 | 94.97 | 60% | 293 | SRS1218630 |
| 6-A31 | 51.7 | 27.1 | 5.1 | 98.57 | 62% | 300 | SRS1218627 |
| 99582-1 | 49.3 | 26.8 | 5.0 | 98.77 | 62% | 297 | SRS1218626 |
| SN-21 | 34.3 | 21.2 | 5.2 | 96.99 | 51% | 268 | SRS1218626 |
| CZB-25 | 40.8 | 26.3 | 5.0 | 97.97 | 62% | 271 | SRS1218625 |
| NL-1217 | 30.5 | 21.1 | 5.1 | 97.05 | 52% | 261 | SRS1218618 |
| FTNY19 | 27.0 | 22.5 | 5.1 | 97.71 | 58% | 281 | SRS1218616 |
| NVHOF3/5 | 30.1 | 22.0 | 5.1 | 97.06 | 55% | 250 | SRS1218592 |
| PG-22 | 39.7 | 32.2 | 4.8 | 98.84 | 58% | 303 | SRS1218624 |

^a^Millions of raw reads obtained for each sample. ^b^Millions of filtered reads of each sample used to create the *P. nigra* pool. ^c^Mean sequencing depth calculated considering only the uniquely aligned selected reads. ^d^Mean read length obtained after quality trimming. ^e^% of *P. trichocarpa* reference genome covered by at least one sequenced read. ^f^Mean library insert size. ^g^Short Read Archive accessions.

**Supplementary Table 3. SRA accessions of *P. trichocarpa* RNAseq libraries.**

| **Run accession** | **Tissue** | **# Reads** |
| --- | --- | --- |
| SRR1121301 | Developing xylem | 12,030,442 |
| SRR1121302 | Developing xylem | 25,506,215 |
| SRR1121303 | Developing xylem | 11,771,045 |
| SRR1030352 | Leaf | 65,442,430 |
| SRR2029745 | Callus from root | 33,887,931 |
| SRR2029746 | Callus from root | 30,706,724 |
| SRR2029776 | Callus from root | 23,909,407 |
| SRR954876 | Cambium | 29,346,207 |
| SRR954877 | Cambium | 27,604,703 |
| SRR954878 | Cambium | 26,546,121 |

**Supplementary Table 4.** Summary statistics of INDELs detected in the high-depth poplar accessions.

| **Accession** | **# DEL^a^** | **Mb DEL^b^** | **Median length DEL** | **Het DEL^c^** | **# INS^d^** | **Mb INS^e^** | **Median length INS** | **Het INS^f^** |
| --- | --- | --- | --- | --- | --- | --- | --- | --- |
| **L155-079** (*P. deltoides*) | 2,853 | 11.6 | 2,243 | 16.8% | 3,673 | 23.8 | 5,241 | 29.3% |
| **L150-089** (*P. deltoides*) | 2,614 | 11.0 | 2,327 | 16.0% | 3,672 | 24.1 | 5,252 | 26.8% |
| **71077-308** (*P. nigra*) | 3,838 | 15.6 | 2,154 | 17.4% | 4,752 | 28.7 | 4,934 | 32.7% |
| **BDG** (*P. nigra*) | 2,444 | 10.6 | 2,320 | 16.4% | 4,686 | 29.5 | 5,119 | 22.0% |
| **Poli** (*P. nigra*) | 3,497 | 15.1 | 2,342 | 14.4% | 4,985 | 30.5 | 4,978 | 22.9% |
| **BEN3** (*P. nigra*) | 2,130 | 8.7 | 2,163 | 20.4% | 4,783 | 29.5 | 4,958 | 17.2% |
| **Nisqually-1** (*P. trichocarpa*) | 1,178 | 4.9 | 2,233 | 91.9% | 707 | 3.8 | 4,629 | 86.3% |
| **Merged results** | 7,889 | 33.2 | 2,176 |  | 10,586 | 62.9 | 4,860 |  |

^a^Number of detected deletions. ^b^Span of deletions in Megabases. ^c^Percentage of heterozygous deletions in each sample. ^d^Number of detected insertions. ^e^Span of insertions in Megabases. ^f^Percentage of heterozygous insertions in each sample.

**Supplementary Table 7.** The table reports the classification of deletions on the bases of the homology with known transposable elements within all deletions (All DEL), *P. nigra* specific deletions (N), *P. deltoides* specific deletions (D), *P. trichocarpa* specific deletions (T) and deletions detected only in *P. nigra* and *P. deltoides* samples (N/D).

|  | **All DEL** | **N** | **D** | **T** | **N/D** |
| --- | --- | --- | --- | --- | --- |
| **Not annotated** | **44.3%** | **51.6%** | **51.1%** | **41.2%** | **22.4%** |
| **LTR Gypsy** | 22.2% | 19.0% | 23.7% | 27.3% | 26.4% |
| **LTR Copia** | 10.8% | 8.4% | 8.7% | 7.0% | 18.9% |
| **LTR Unknown** | 0.5% | 0.5% | 0.2% | 0.3% | 0.7% |
| **LINE L1** | 0.8% | 0.7% | 0.5% | 1.7% | 0.7% |
| **SINE** | 0.1% | 0.1% | 0.2% | 0.0% | 0.0% |
| **Total class I** | **34.4%** | **28.7%** | **33.3%** | **36.3%** | **46.8%** |
| **TIR hAT** | 6.7% | 5.6% | 4.7% | 7.5% | 10.5% |
| **Helitron** | 4.8% | 5.2% | 3.1% | 5.3% | 5.8% |
| **TIR CACTA** | 4.3% | 4.4% | 4.2% | 3.8% | 4.9% |
| **TIR PIF/Harbinger** | 0.5% | 0.4% | 0.3% | 0.3% | 1.4% |
| **TIR Mutator** | 0.3% | 0.4% | 0.4% | 0.2% | 0.1% |
| **DNA Unknown** | 4.6% | 3.8% | 2.8% | 5.3% | 8.1% |
| **Total class II** | **21.3%** | **19.7%** | **15.6%** | **22.4%** | **30.8%** |

**Supplementary Table 8.** The table reports the classification of insertions on the bases of the homology with known transposable elements for all insertions (All INS), *P. nigra* specific insertions (N), *P. deltoides* specific insertions (D), *P. trichocarpa* specific insertions (T) and insertions detected only in *P. nigra* and *P. deltoides* samples (N/D)

|  | **All INS** | **N** | **D** | **T** | **N/D** |
| --- | --- | --- | --- | --- | --- |
| **Not annotated** | 1.8% | 1.6% | 1.9% | 3.1% | 1.9% |
| **LTR Gypsy** | 48.9% | 42.5% | 45.4% | 33.9% | 81.7% |
| **LTR Copia** | 27.0% | 31.7% | 29.2% | 25.5% | 8.0% |
| **LTR Unknown** | 2.9% | 3.0% | 3.5% | 3.6% | 1.6% |
| **LINE L1** | 3.4% | 3.8% | 2.7% | 4.2% | 3.0% |
| **SINE** | 0.0% | 0.0% | 0.0% | 0.0% | 0.0% |
| **Total class I** | **82.3%** | **81.0%** | **80.8%** | **67.2%** | **94.2%** |
| **TIR hAT** | 4.2% | 5.1% | 3.7% | 8.0% | 1.0% |
| **Helitron** | 2.4% | 3.0% | 2.0% | 2.9% | 1.0% |
| **TIR CACTA** | 4.5% | 4.5% | 6.1% | 7.1% | 1.2% |
| **TIR PIF/Harbinger** | 0.9% | 1.1% | 0.8% | 1.5% | 0.2% |
| **DNA Unknown** | 3.9% | 3.8% | 4.7% | 10.2% | 0.5% |
| **Total class II** | **15.9%** | **17.5%** | **17.2%** | **29.7%** | **3.9%** |

**Supplementary Table 10.** Summary of detection of genic CNVs. **Genic CNVs**: Number of genic CNVs identified in pairwise comparisons. **S1 > S2**: number of genic CNVs showing higher copy number in Sample1 compared to Sample2. **S2 > S1**: number of genic CNVs showing higher copy number in Sample2 compared to Sample1.

| **Sample1** | **Species1** | **Sample2** | | **Species2** | | **Genic CNVs** | | **S1 > S2** | | **S1 < S2** | |  |
| --- | --- | --- | --- | --- | --- | --- | --- | --- | --- | --- | --- | --- |
| L150-089 | *P. deltoides* | | L155-079 | | *P. deltoides* | | 747 | | 417 | | 330 | |
| Poli | *P. nigra* | | 71077-308 | | *P. nigra* | | 747 | | 387 | | 360 | |
| 71077-308 | *P. nigra* | | L150-089 | | *P. deltoides* | | 1192 | | 512 | | 680 | |
| 71077-308 | *P. nigra* | | L155-079 | | *P. deltoides* | | 1162 | | 540 | | 622 | |
| Poli | *P. nigra* | | L150-089 | | *P. deltoides* | | 1213 | | 551 | | 662 | |
| Poli | *P. nigra* | | L155-079 | | *P. deltoides* | | 1174 | | 556 | | 618 | |
| 71077-308 | *P. nigra* | | Nisqually | | *P. trichocarpa* | | 1067 | | 248 | | 819 | |
| Poli | *P. nigra* | | Nisqually | | *P. trichocarpa* | | 1103 | | 286 | | 817 | |
| L150-089 | *P. deltoides* | | Nisqually | | *P. trichocarpa* | | 1154 | | 323 | | 831 | |
| L155-079 | *P. deltoides* | | Nisqually | | *P. trichocarpa* | | 1108 | | 322 | | 786 | |

**Supplementary Table 13. Genes involvement in INDELs.** The table reports the Megabases (Mb) annotated as five prime untranslated regions (5’ UTR), three prime untranslated regions (3’ UTR), coding regions (CDS) and intronic regions (Intron) in the *P. trichocarpa* reference genome (REF) in the regions affected by deletions (DEL), and in a simulated set of deletions (Expected DEL). For genes interrupted by insertions, the table reports the number of insertions predicted in each of the four different genic regions in the experimental sample (INS) and in a simulated dataset (Expected INS).

|  | **REF (Mb)** | **DEL (Mb)** | **Expected DEL (Mb)** | **INS (#)** | **Expected INS (#)** |
| --- | --- | --- | --- | --- | --- |
| **5' UTR** | 5.0 | 0.09 | 0.40 (0.38-0.43) | 75 | 56 (38-76) |
| **3' UTR** | 8.9 | 0.19 | 0.71 (0.68-0.75) | 218 | 119 (99-140) |
| **CDS** | 47.9 | 1.76 | 3.82 (3.70-3.93) | 507 | 642 (598-690) |
| **Intron** | 62.4 | 2.37 | 5.14 (4.95-5.36) | 807 | 1045 (995-1103) |

**Supplementary Table 14. Library statistics of the three simulated datasets.**

| **Dataset description** | **Read length (bp)** | **Insert size (bp)** | **Genome covered** | **Mean depth** |
| --- | --- | --- | --- | --- |
| Real Nisqually-1 reads | 75 | 225 | 92% | 41.2X |
| Simulated reads from the original reference | 100 | 420 | 93% | 31.2X |
| Simulated reads from the original reference plus simulated reads from the modified reference | 100 | 420 | 97% | 32.8X |

**Supplementary Table 15**. Simulation results for the detection of deletions using the real dataset.

|  | **CLEVER** | **DELLY** | **GASV** | **Pindel** | **DELLY**  **CLEVER** | **DELLY**  **GASV** | **DELLY**  **Pindel** |
| --- | --- | --- | --- | --- | --- | --- | --- |
| **# predictions** | 1,238 | 1,755 | 1,773 | 1,732 | 1842 | **1819** | 2121 |
| **# true positives** | 639 | 820 | 831 | 774 | 840 | **837** | 859 |
| **# false negatives** | 361 | 180 | 169 | 226 | 160 | **163** | 141 |
| **# false positives** | 599 | 935 | 942 | 958 | 1002 | **982** | 1262 |
| **Sensitivity** | 63.9% | 82.0% | 83.1% | 77.4% | 84.0% | **83.7%** | 85.9% |
| **Precision** | 51.62% | 46.72% | 46.87% | 44.69% | 45.60% | **46.01%** | 40.50% |
| **Breakpoint distance (bp)** | 8.0 | 3.0 | 7.9 | 1.9 | 3,54 | **3,34** | 2,96 |
| **# of exact predictions** | 90 | 767 | 83 | 759 | 770 | **769** | 803 |

**Supplementary Table 16**. Simulation results for the detection of deletions using the simulated dataset.

|  | **CLEVER** | **DELLY** | **GASV** | **Pindel** | **DELLY**  **CLEVER** | **DELLY**  **GASV** | **DELLY**  **Pindel** |
| --- | --- | --- | --- | --- | --- | --- | --- |
| **# predictions** | 847 | 937 | 860 | 846 | 957 | **958** | 947 |
| **# true positives (TP)** | 846 | 936 | 859 | 831 | 955 | **957** | 940 |
| **# false negatives (FN)** | 154 | 64 | 141 | 169 | 45 | **43** | 60 |
| **# false positives (FP)** | 1 | 1 | 1 | 15 | 2 | **1** | 7 |
| **Sensitivity** | 84.6 % | 93.6% | 85.9% | 83.1% | 95.5% | **95.7%** | 94.0% |
| **False discovery rate (FDR)** | 0.12% | 0.11% | 0.12% | 1.77% | 0.21% | **0.10%** | 0.74% |
| **Precision** | 99.88% | 99.89% | 99.88% | 98.23% | 99.79% | **99.90%** | 99.26% |
| **Breakpoint distance (bp)** | 12.9 | 2.8 | 45.4 | 1.8 | 3.51 | **5.21** | 3.02 |
| **# of exact predictions** | 67 | 889 | 20 | 829 | 889 | **889** | 893 |

**Supplementary Table 17**. Simulation results for the detection of insertions using the real and the simulated dataset.

|  | **Real dataset** | **Simulated dataset** |
| --- | --- | --- |
| **# predictions** | 1230 | 877 |
| **TPs** | 766 | 877 |
| **FNs** | 234 | 123 |
| **FPs** | 374 | 0 |
| **Sensitivity** | 76.60% | 87.70% |
| **FDR** | n.a. | 0 |
| **Precision** | 62.30% | 100.00% |
| **Accuracy** | 5 | 25.3 |
